# Deep-Learning for Epicardial Adipose Tissue Assessment With Computed Tomography: Implications for Cardiovascular Risk Prediction

**DOI:** 10.1016/j.jcmg.2022.11.018

**Published:** 2023-06

**Authors:** Henry W. West, Muhammad Siddique, Michelle C. Williams, Lucrezia Volpe, Ria Desai, Maria Lyasheva, Sheena Thomas, Katerina Dangas, Christos P. Kotanidis, Pete Tomlins, Ciara Mahon, Attila Kardos, David Adlam, John Graby, Jonathan C.L. Rodrigues, Cheerag Shirodaria, John Deanfield, Nehal N. Mehta, Stefan Neubauer, Keith M. Channon, Milind Y. Desai, Edward D. Nicol, David E. Newby, Charalambos Antoniades, ORFAN Investigators

**Affiliations:** a Acute Multidisciplinary Imaging and Interventional Centre, Radcliffe Department of Medicine, University of Oxford, Oxford, United Kingdom; b Division of Cardiovascular Medicine, Radcliffe Department of Medicine, University of Oxford, Oxford, United Kingdom; c Caristo Diagnostics Pty Ltd, Oxford, United Kingdom; d Centre for Cardiovascular Science, University of Edinburgh, Edinburgh, Scotland, United Kingdom; e Northwestern University, Evanston, Illinois, USA; f Royal Brompton and Harefield National Health Service (NHS) Foundation Trust, London, United Kingdom; g Translational Cardiovascular Research Group, Department of Cardiology, Milton Keynes University Hospital, Milton Keynes, United Kingdom; h Faculty of Medicine and Health Sciences, University of Buckingham, Buckingham, United Kingdom; i Department of Cardiovascular Sciences and National Institute for Health Research Leicester Biomedical Research Centre, University of Leicester, Leicester, United Kingdom; j Royal United Hospitals Bath NHS Foundation Trust, Bath, United Kingdom; k Department of Health, University of Bath, Bath, United Kingdom; l Department of Cardiology, Oxford University Hospitals NHS Foundation Trust, Oxford, United Kingdom; m University College London, London, United Kingdom; n National Heart, Lung, and Blood Institute, National Institutes of Health, Bethesda, Maryland, USA; o The Cleveland Clinic, Cleveland, Ohio, USA; p School of Biomedical Engineering and Imaging Sciences, King’s College London, London, United Kingdom

**Keywords:** adipose tissue, atherosclerosis, atrial fibrillation, computed tomography, deep-learning, visceral fat

## Abstract

**Background:**

Epicardial adipose tissue (EAT) volume is a marker of visceral obesity that can be measured in coronary computed tomography angiograms (CCTA). The clinical value of integrating this measurement in routine CCTA interpretation has not been documented.

**Objectives:**

This study sought to develop a deep-learning network for automated quantification of EAT volume from CCTA, test it in patients who are technically challenging, and validate its prognostic value in routine clinical care.

**Methods:**

The deep-learning network was trained and validated to autosegment EAT volume in 3,720 CCTA scans from the ORFAN (Oxford Risk Factors and Noninvasive Imaging Study) cohort. The model was tested in patients with challenging anatomy and scan artifacts and applied to a longitudinal cohort of 253 patients post-cardiac surgery and 1,558 patients from the SCOT-HEART (Scottish Computed Tomography of the Heart) Trial, to investigate its prognostic value.

**Results:**

External validation of the deep-learning network yielded a concordance correlation coefficient of 0.970 for machine vs human. EAT volume was associated with coronary artery disease (odds ratio [OR] per SD increase in EAT volume: 1.13 [95% CI: 1.04-1.30]; *P =* 0.01), and atrial fibrillation (OR: 1.25 [95% CI: 1.08-1.40]; *P =* 0.03), after correction for risk factors (including body mass index). EAT volume predicted all-cause mortality (HR per SD: 1.28 [95% CI: 1.10-1.37]; *P =* 0.02), myocardial infarction (HR: 1.26 [95% CI:1.09-1.38]; *P =* 0.001), and stroke (HR: 1.20 [95% CI: 1.09-1.38]; *P =* 0.02) independently of risk factors in SCOT-HEART (5-year follow-up). It also predicted in-hospital (HR: 2.67 [95% CI: 1.26-3.73]; *P ≤* 0.01) and long-term post–cardiac surgery atrial fibrillation (7-year follow-up; HR: 2.14 [95% CI: 1.19-2.97]; *P ≤* 0.01).

**Conclusions:**

Automated assessment of EAT volume is possible in CCTA, including in patients who are technically challenging; it forms a powerful marker of metabolically unhealthy visceral obesity, which could be used for cardiovascular risk stratification.

Coronary computed tomography angiography (CCTA) is used to evaluate coronary artery disease (CAD) risk,^1^ with guidelines in Europe^2^ and the United States^3^ recommending CCTA for assessment of patients with chest pain. The use of CCTA for detecting CAD is increasing worldwide, and it is likely that further valuable information within CCTA scans that is currently not fully utilized in clinical practice could improve risk assessment and patient management for cardiometabolic diseases, with multiple such technologies being discovered through the uptake of artificial intelligence methods in research and practice.^4^

Adipose tissue is recognized as a key regulator of cardiovascular health and disease, exerting both protective and deleterious effects on the cardiovascular system.^5^ Epicardial adipose tissue (EAT) is a metabolically active depot of visceral fat^5^ and may be a feature of metabolically unhealthy obesity and metabolic syndrome.^6^ Indeed, EAT volume has been associated with multiple distinct cardiovascular diseases including CAD and atrial fibrillation (AF).^7^ The EAT volume is generally considered to be a marker of visceral obesity, as opposed to more sophisticated metrics such as the pericoronary Fat Attenuation Index, which specifically captures the degree of coronary inflammation, and has prognostic value over and above that of EAT volume.^8^ CCTA provides the noninvasive gold standard measurement of EAT volume because of its excellent spatial resolution. However, manual quantification is laborious and currently falls outside the scope of routine CCTA interpretation. If clinical utility of automated EAT volume quantification could be demonstrated and found to be feasible in patients with technically challenging CCTA, it is possible that this measure could become part of standard of care.

In this study we developed and validated a deep-learning network (DLN) for the automated quantification of EAT volume, which was then tested in real-world patients with CCTA with commonly encountered image quality issues to ensure validity. Then, we applied the fully automated EAT quantification tool to investigate the clinical association of EAT volume with relevant cross-sectional and longitudinal disease outcomes (Central Illustration).

**Central Illustration:**
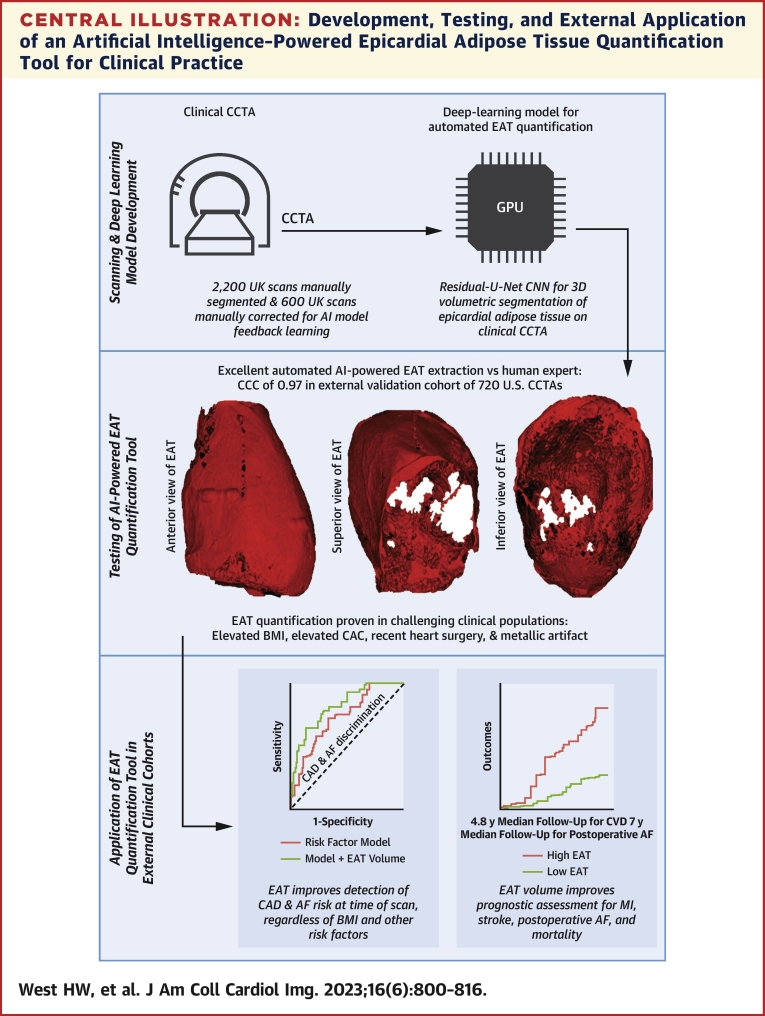
Development, Testing, and External Application of an Artificial Intelligence–Powered Epicardial Adipose Tissue Quantification Tool for Clinical Practice **(Top)** A deep-learning model was trained to automatically extract the adipose tissue from CCTA. **(Middle)** The model performed excellently compared to human segmentation in internal and external testing, including in patient groups that are commonly occurring yet challenging for CCTA. **(Bottom)** The final automated artificial intelligence (AI) model for epicardial adipose tissue (EAT) quantification was applied to external clinical cohorts and revealed improved detection of prevalent disease risk for coronary artery disease (CAD) and atrial fibrillation (AF) and provided incremental prognostic benefit for key cardiovascular events such as myocardial infarction (MI), stroke, postoperative AF, and mortality in longitudinal cohorts. 3D = 3-dimensional; BMI = body mass index; CAC = coronary artery calcium; CCC = Lin concordance correlation coefficient; CNN = convolutional neural network; CCTA = coronary computed tomography angiography; CVD = cardiovascular disease; GPU = graphics processing unit.

## Methods

### Study populations

**Figure 1:**
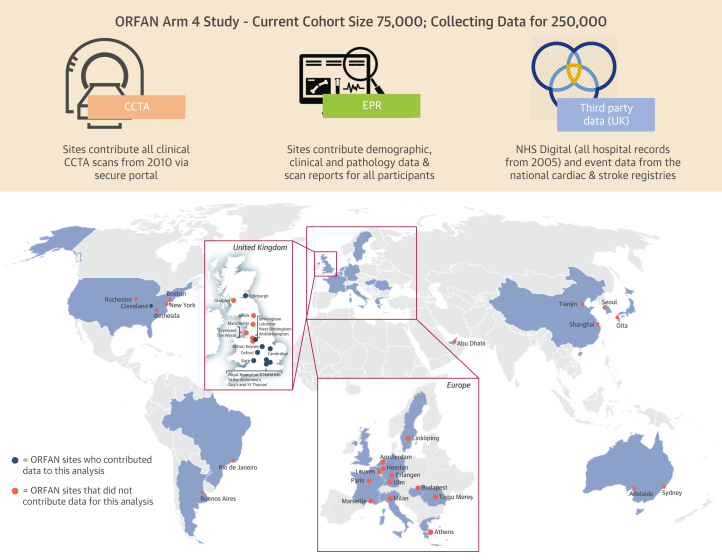
The ORFAN Arm 4 Study The ORFAN (Oxford Risk Factors and Noninvasive Imaging Study) Arm 4 study is an international multicenter retrospective cohort study of patients undergoing clinically indicated CCTA. The initial cohort size is 75,000 patients within the United Kingdom and 25,000 internationally, with ethically approved expansion underway for 250,000 patients. Within the United Kingdom, the study includes 17 National Health Service (NHS) Trusts, 4 of which contributed data to the current study. Data collected for each participant includes the CCTA scan, data from the local hospital electronic patient record (EPR), and data from authorized third parties, including NHS Digital, all hospital event data from 2005 to now; NICOR (National Institute for Cardiovascular Outcomes Research), a national cardiac event registry; and SSNAP (Sentinel Stroke National Audit Programme), a national stroke event registry. CCTA = coronary computed tomography angiography.

Each study (ORFAN [Oxford Risk Factors and Noninvasive Imaging Study], AdipoRedOx [Adipose tissue and cardiovascular RedOx regulation] study, and the SCOT-HEART [Scottish Computed Tomography of the Heart] trial) received ethical approval. The full ethics, population descriptions, variable definitions, laboratory techniques, and CCTA acquisition and retrieval protocols are outlined in the Supplemental Methods. Briefly, the ORFAN study (NCT05169333) is an international multicenter prospective cohort study that collects CCTA scans and patient clinical data from those who are undergoing or have undergone CCTA since 2005.^9^ For this analysis, data were utilized from across 4 National Health Service sites in England and 1 in the United States (Figure 1). The AdipoRedOx study involves patients who are undergoing cardiac surgery; as part of the study, patients undergo CCTA shortly after their operation and are prospectively followed up for clinical outcomes via National Health Service's NHS Digital (see the Supplemental Methods). The SCOT-HEART trial included clinical patients with suspected angina caused by coronary heart disease, who were followed up for 5 years post-CCTA for clinical outcomes (see the Supplemental Methods).

### Overall study design

**Figure 2:**
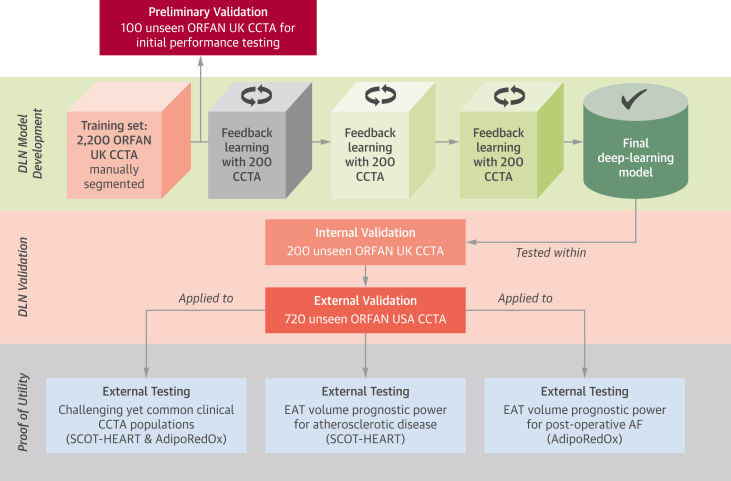
Study Flowchart of Model Development, Testing, and External Application Schematic representation of the scientific approach to the development of the deep-learning model, the validation of the model through internal and external cohorts, and the application of the automated epicardial adipose tissue (EAT) volume quantification tool to 3 groups of patients from the AdipoRedOx (Adipose tissue and cardiovascular RedOx regulation) study and SCOT-HEART (Scottish Computed Tomography of the Heart) trial. AF = atrial fibrillation; DLN = deep-learning network; other abbreviations as in Figure 1.

The overall approach to the development of the DLN, the internal and external validation, and the application of the DLN in external cohorts for ascertainment of clinical utility is outlined in Figure 2. In summary, 2,200 CCTA scans from the ORFAN study were used for training the DLN for the detection of the whole heart within the bounds of the pericardium. Following training, an initial assessment of model performance was performed in 100 unseen ORFAN study scans (Supplemental Figures 1 and 2). Three separate groups of 200 unseen scans from the ORFAN study were used for fine-tuning the model through 3 iterations of feedback learning.

The DLN was tested internally on 200 unseen ORFAN study scans from the UK sites of the study. External validation was performed on 720 unseen scans from the U.S. sites of the ORFAN study. The DLN was then applied to unseen scans from challenging clinical populations to test the model in patients with challenging anatomy and/or commonly occurring scan artifacts. Finally, the model was tested in unseen external scans of the SCOT-HEART trial for real-world evaluation of the prognostic value of EAT volume as a marker of metabolically unhealthy obesity. The model was also applied within the AdipoRedOx study to test the prognostic value of EAT volume on the risk of in-patient post–cardiac surgery AF (>30 seconds of AF on monitoring) and long-term AF (paroxysmal, persistent, or chronic) following surgery were investigated.

### Developing the DLN for automated segmentation and quantification of EAT volume

**Figure 3:**
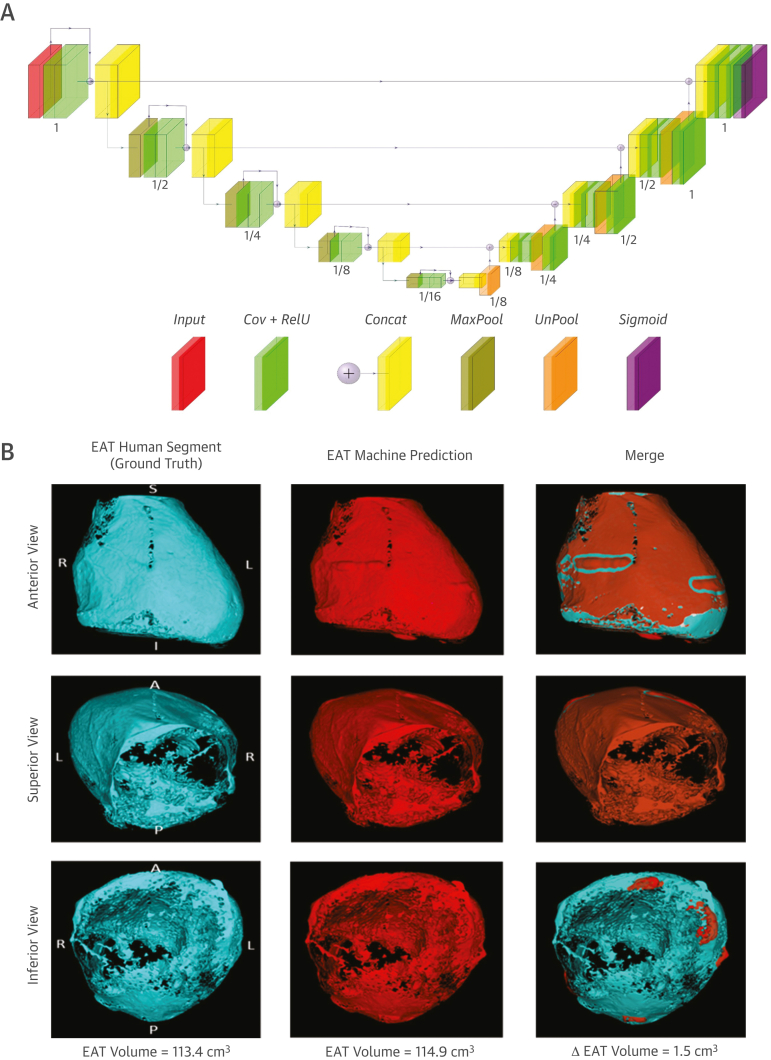
Schematic of the Deep-Learning Model for Automated Segmentation of the Whole Heart Within the Pericardium and Example Automated Segmentation **(A)** Block diagram of the Residual U-Net–based convolutional neural network architecture. Details of each layer are provided in the Supplemental Methods. **(B)** A single CCTA from the ORFAN study demonstrating human expert segmentation as ground truth, automated machine segmentation and a merge. A = anterior; Concat = concatenation; Cov = convolution; I = inferior; L = left; P = posterior; R = right; RelU = rectified linear activation function; S = superior; other abbreviations as in Figures 1 and 2.

Manual segmentation of the 2,200 CCTA and the iterations of scans for feedback learning of the DLN, and the automated extraction of EAT volume from the heart segmentation were performed using CaRi-Heart (version 2.2.1, Caristo Diagnostics Ltd) (Supplemental Figure 3).^10^ A fully automated method for whole heart segmentation on CCTA scans was employed using a 3-dimensional Residual-U-Net neural network architecture for volumetric segmentation of CCTA (Supplemental Methods). The architecture of the DLN is demonstrated in Figure 3A.

### Internal validation

A random sample of 200 sequestered CCTA from the UK sites in the ORFAN study were used for internal validation of the DLN. Human segmentation of these scans was undertaken blind to all other data.

### External validation

A sample of 720 unseen CCTA from the Cleveland Clinic site of the ORFAN study were used for external validation of the algorithm—as a broad external validation cohort.^11^ The manual segmentation of these scans was undertaken blind to all other data.

### Statistical analysis

For inter-reader repeatability testing and human vs automated model assessment of testing data (preliminary testing, internal/external validation, and challenging clinical populations) agreement was assessed by using the Lin concordance correlation coefficients (CCC) with scatterplots and Bland-Altman analysis for significance of bias. When applied in a cohort-wide setting in the AdipoRedOx and SCOT-HEART studies, all EAT volumes were standardized by patient body surface area using the Du Bois formula.

For cross-sectional analysis of disease risk conveyed by EAT volume, multiple-adjusted logistic regression was used for calculation of the odds ratio of prevalent disease (AF at time of CCTA and obstructive CAD from CCTA) at the time of the CCTA given increase in EAT by 1 SD. All analyses were adjusted for a standard set of cardiovascular disease (CVD) risk factors that are listed within all figure legends. Longitudinal assessment of the prognostic value of EAT volume was performed by multivariable Cox regression models adjusted for the standard set of clinical risk factors for each outcome. Both odds ratios and HRs are reported per SD increase in EAT volume. Analysis of all SCOT-HEART trial risk models was repeated with the inclusion of only variables in each model that were found to have a statistically meaningful association with the relevant outcome (dependent variable) in univariate analysis, at the level of *P ≤* 0.1 (Supplemental Methods, Supplemental Table 1). Supplemental analysis of AdipoRedOx study risk models was performed with AF-specific risk factors (Supplemental Methods, Supplemental Figure 4).

Receiver-operating characteristic curves for the discrimination of obstructive CAD from CCTA and myocardial infarction (MI) were plotted with a CVD risk factor models vs traditional risk factor model plus the addition of EAT volume. Area under the curve analysis was undertaken to compare the models for each outcome.

We selected the optimum unified cutoff for EAT volume prognostication for all-cause mortality, fatal/nonfatal MI, and fatal/nonfatal stroke in SCOT-HEART by identifying the value that maximized the Youden J statistic (sum of sensitivity and specificity) on time-dependent receiver-operating characteristic curve analysis for all-cause mortality, fatal/nonfatal MI, and fatal/nonfatal stroke to ensure an optimum balance between sensitivity and specificity in our models. For consistency, the same approach was used to select the optimum cutoff for EAT volume prognostication for in-hospital postoperative and long-term AF risk within the severe CAD population of the AdipoRedOx study. Further discussion about the statistical analysis is in the Supplemental Methods.

## Results

The geographic location, demographics, clinical risk factors, and CCTA scan technical parameters for all ORFAN study cohorts used in DLN training, validation, and external testing are shown in Table 1. The demographics and scan characteristics of the external clinical cohorts for which the DLN were applied following development are shown in Table 2. The relevant clinical outcomes for the prospective clinical cohorts are presented in Supplemental Table 2. In all cohorts where EAT volumes were quantified, both manually and automatically, the values were normally distributed.

**Table 1:** Demographics and Scan Characteristics of ORFAN Study Cohorts Used in AI Model Development and Testing

	ORFAN Manual Training Cohort (n = 2,200)	ORFAN UK Feedback Training Cohort (n = 600)	ORFAN UK Internal Validation Cohort (n = 200)	ORFAN USA External Validation Cohort (n = 720)
Age, y	60 (50-70)	57 (49-64)	56 (48-62)	53 (43-62)
Male	1,047 (47.6)	315 (52.5)	104 (52.0)	389 (54.0)
BMI, kg/m^2^	26.6 (23.6-29.9)	26.1 (23.1-30.0)	27.7 (24.8-30.7)	27.9 (24.5-32.2)
Active smoking	376 (17.0)	93 (15.5)	27 (13.5)	NA
Hypertension	711 (32.0)	228 (38.0)	73 (36.5)	309 (42.9)
Hypercholesterolemia	896 (40.7)	253 (42.2)	76 (38.0)	370 (51.4)
Diabetes mellitus	286 (13.0)	101 (16.8)	25 (12.5)	73 (10.1)
Valve disease	139 (6.3)	31 (5.2)	13 (6.5)	49 (6.8)
Known CAD	188 (8.5)	55 (9.2)	21 (10.5)	331 (46.0)
Atrial fibrillation	98 (4.5)	21 (3.5)	9 (4.5)	98 (13.6)
Previous heart surgery	47 (2.1)	19 (3.2)	7 (3.5)	16 (2.2)
Scans				
Sites (scanner make and model)	Oxford, UK (GE Revolution GSI, GE Lightspeed VCT, and Canon Aquilion One) Bath, UK (Siemens Drive) Milton Keynes, UK (Canon Aquilion Prime SP) Leicester, UK (Siemens Definition Flash)		Oxford, UK (GE Revolution GSI, GE Lightspeed VCT, and Canon Aquilion One)	Cleveland, Ohio, USA (Philips Brilliance iCT and Siemens Definition Flash)
Tube voltage, kVp				
120	2,112 (96.0)	567 (94.5)	193 (96.5)	463 (64.3)
100	81 (3.7)	33 (5.5)	7 (3.5)	257 (35.7)
80	7 (0.3)	0 (0)	0 (0)	0 (0)
EAT volume, cm^3^	133.2 (100.1-191.8)	124.9 (97.4-203.2)	120.9 (95.1-156.2)	169.3 (111.6-241.7)

Values are n (%) or median (IQR).

AI = artificial intelligence; BMI = body mass index; CAD = coronary artery disease; EAT = epicardial adipose tissue; GE = General Electric; NA = not applicable; ORFAN = Oxford Risk Factors and Noninvasive Imaging Study.

**Table 2:** Demographics and Scan Characteristics of External Clinical Cohorts

	AdipoRedOx Cohort (n = 253)	SCOT-HEART Cohort (n = 1,558)
Age, y	67 (59-74)	58 (47-69)
Male	220 (87.0)	887 (56.9)
BMI, kg/m^2^	27.9 (25.0-31.1)	28.7 (25.1-33.2)
Active smoking	127 (50.2)	298 (19.1)
Hypertension	186 (73.5)	541 (34.7)
Hypercholesterolemia	227 (89.7)	810 (52.0)
Diabetes mellitus	55 (21.7)	173 (11.1)
Valve disease	48 (19.0)	144 (9.2)
Known CAD	253 (100)	158 (10.1)
Atrial fibrillation	18 (7.1)	32 (2.1)
Previous heart surgery	253 (100)	33 (2.1)
Scans		
Site	Oxford GE Revolution GSI GE Lightspeed VCT Canon Aquilion One	Scotland Philips Brilliance 64 Siemens Biograph mCT Canon Aquilion One
Scanner make and model		
Tube voltage, kVp		
120	253 (100)	846 (54.3)
100	0 (0)	712 (45.7)
80	0 (0)	0 (0)
EAT volume, cm^3^	162.9 (103.3-223.3)	130.1 (94.2-171.6)

Values are n (%) or median (IQR).

AdipoRedOx = Adipose tissue and cardiovascular RedOx regulation; SCOT-HEART = Scottish Computed Tomography of the Heart; other abbreviations as in Table 1.

### Inter-reader repeatability for EAT segmentation and whole heart segmentation

The interobserver variability between 2 expert analysts of the Oxford Academic Cardiovascular Computed Tomography Core Lab core lab was evaluated in 100 randomly selected patients from the UK sites of the ORFAN study. CCC for EAT volume was excellent between readers at 0.970, and the bias was nonsignificant at mean of 2.1 (95% agreement: −3.9 to 6.1) cm^3^ (*P =* 0.74) (Supplemental Figures 5A and 5B). For the whole heart segmentation volume, CCC was also excellent at 0.969 with nonsignificant bias mean of 15.2 (95% agreement: −7.5 to 23.1) cm^3^ (*P =* 0.08) (Supplemental Figures 5C and 5D).

### Internal validation of the model

**Figure 4:**
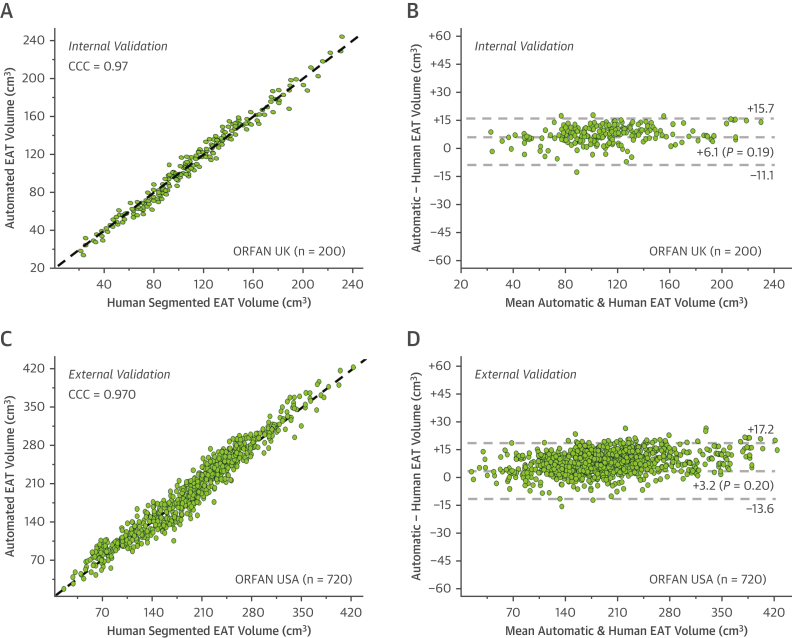
Validation of the Deep-Learning Model Following all training and fine-tuning of the algorithm, internal validation in 200 ORFAN UK cases occurred and is demonstrated in the scatterplot **(A)** and Bland-Altman plot **(B).** External validation in 720 ORFAN USA scans is shown in the scatterplot **(C)** and Bland-Altman plot **(D).** CCC = Lin concordance correlation coefficient; other abbreviations as in Figures 1 and 2.

Final internal validation occurred following 3 iterations of feedback learning to enhance the performance of the model. The median EAT volume in internal validation was 120.9 (IQR: 95.1-156) cm^3^. When applied to 200 unseen scans from the UK sites of the ORFAN study, the CCC was 0.972 (Figure 4A). The bias in Bland-Altman analysis (Figure 4B) was also nonsignificant at 6.1 (IQR: −11.1 to 15.7) cm^3^ (*P =* 0.19).

### External validation of the model

The final deep-learning model was applied to 720 unseen scans from the U.S. sites of the ORFAN study. The mean automated analysis time for the automated segmentation was 12.4 seconds compared with mean manual segmentation time of 18 minutes and 20 seconds. The median EAT volume in external validation was 169.3 (IQR: 111.6-241.7) cm^3^. The CCC for the automated deep-learning model vs human expert segmentation in the external validation cohort was excellent, at 0.970 (Figure 4C) and the bias in Bland-Altman analysis (Figure 4D) was nonsignificant at 3.2 (IQR: −13.6 to 17.2) cm^3^ (*P =* 0.20).

### Validation of the automated model for EAT volume quantification in challenging clinical populations

**Figure 5:**
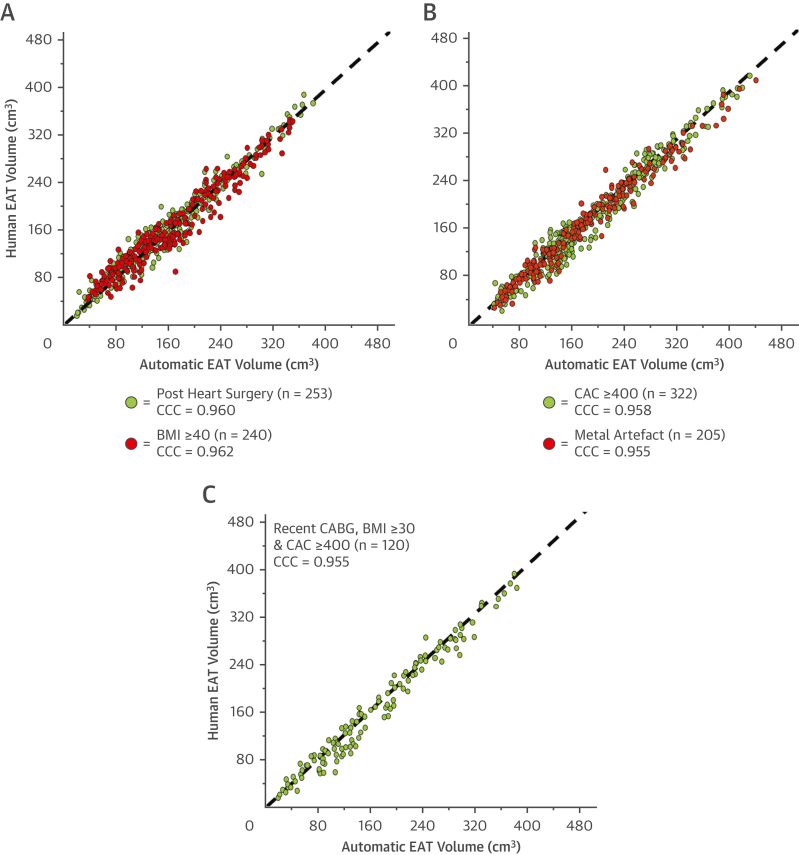
Validation of the Automated Deep-Learning Model in Challenging Clinical Populations The automated EAT volume quantification tool was applied to groupings of unseen CCTA from the AdipoRedOx study and the SCOT-HEART trial. **(A)** Patients who underwent open heart surgery, specifically coronary artery bypass graft (CABG), up to 6 weeks prior to CCTA **(green)** and patients with body mass index (BMI) ≥40 kg/m^2^**(red)**; **(B)** patients with coronary artery calcium (CAC) ≥400 **(green)** and patients with significant metallic artifact within the pericardium **(red)**; **(C)** patients who underwent open heart surgery (CABG) up to 6 weeks prior to the CCTA, had BMI ≥30 kg/m^2^ and CAC ≥400. Abbreviations as in Figures 1, 2, and 4.

Excellent CCC for automated EAT segmentation vs human expert segmentation was achieved in all challenging patient groups: Patient’s with recent cardiac surgery (<6 weeks post operation) CCC = 0.960 (Figure 5A, green); patients with body mass index (BMI) ≥40, CCC = 0.962 (Figure 5A, red); patients with reported coronary artery calcium (CAC) score of ≥400 (Figure 5B, green), CCC = 0.958; patients with significant metallic artifact within the pericardium, CCC = 0.955 (Figure 5B, red), and a combined patient group of recent open-heart surgery, BMI ≥30 kg/m^2^ and CAC ≥400, CCC = 0.955 (Figure 5C).

### Cross-sectional clinical correlations

At the time of the CCTA, application of the fully automated segmentation tool for quantification of EAT volume was found to be a significant independent predictor of the presence of AF at time of CCTA and obstructive CAD from CCTA (any 1 coronary vessel with ≥50% stenosis on CCTA), within 1,558 patients randomized to receive CCTA in the SCOT-HEART trial population.

**Figure 6:**
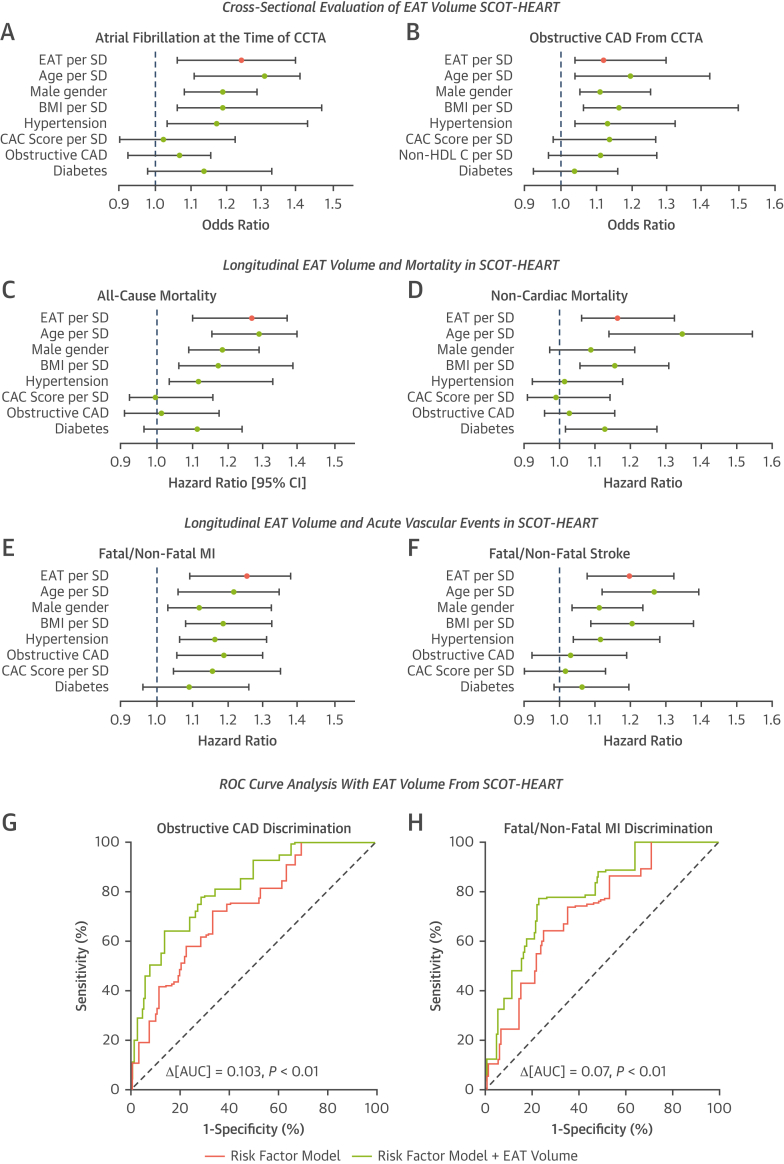
Cross-Sectional and Longitudinal Associations Between EAT Volume and Clinical Outcomes in the SCOT-HEART Trial Plots of cross-sectional adjusted risk models for atrial fibrillation at the time of the scan, adjusted for age, sex, BMI, hypertension, CAC score, diabetes, and obstructive coronary artery disease (CAD) as detected on CT **(A)**; and obstructive CAD (any 1 coronary vessel with ≥50% stenosis on CCTA), adjusted for the same risk factors plus non-HDL cholesterol and without obstructive CAD **(B).** Odds ratio is shown per 1-SD increase in EAT volume for 1,558 patients randomized to receive CCTA in the SCOT-HEART trial. Plots of longitudinal HRs per SD increase in EAT volume in 1,558 patients randomized to receive CCTA in the SCOT-HEART trial are shown for all-cause mortality **(C)** and noncardiac mortality **(D),** both with the same adjustment as for **A**. MI (both fatal and nonfatal) **(E)** and stroke (both fatal and nonfatal) **(F)** are shown with the same adjustment. Receiver-operating characteristic (ROC) curves are shown for the discrimination of obstructive CAD at the time of the CCTA **(G)** and longitudinal MI **(H).** The risk factor model **(green)** for each curve includes age, male sex, BMI, hypertension, non-HDL cholesterol, diabetes, and CAC score, with obstructive CAD also included in the MI model. **Red** in both curve is the risk factor model with the addition of EAT volume. ∗Continuous variables per SD increase; †*P <* 0.05. AUC = area under the curve; CT = computed tomography; HDL = high-density lipoprotein; MI = myocardial infarction; other abbreviations as in Figures 1, 2, and 5.

When accounting for CVD risk factors the odds ratio of AF at time of CCTA per SD increase of EAT was 1.25 (95% CI: 1.08-1.40; *P =* 0.03) (Figure 6A). When accounting for CVD risk factors the odds ratio of obstructive CAD from the CCTA per SD increase of EAT was 1.13 (95% CI: 1.04-1.30; *P =* 0.01) (Figure 6B). Results with statistically selected risk factor adjustment is shown in Supplemental Figure 4.

### Longitudinal EAT volume clinical correlations

Median follow-up for the 1,558 patients randomized to receive CCTA in the SCOT-HEART trial, which were analyzed was 4.8 years. There were 35 deaths of all causes (2.25%) of which 4 were related to coronary heart disease (0.25%). There were 8 fatal and nonfatal strokes (0.51%) and 39 fatal and nonfatal myocardial infarctions (2.5%).

The HR of all-cause mortality per SD increase of EAT was 1.28 (95% CI: 1.10-1.37); *P =* 0.02, after accounting for CVD risk factors (Figure 6C). When adjusted for the same risk factors, the HR of noncardiac mortality per SD increase of EAT volume was 1.17 (95% CI: 1.07-1.33; *P =* 0.04) (Figure 6D). This constitutes a ΔHR of −0.10, confirming that EAT is a measure of visceral adipose tissue related to multiple fatal pathologies, unrelated to CAD. When accounting for CVD risk factors, the HR of MI per SD increase of EAT was 1.26 (95% CI: 1.09-1.38; *P =* 0.001) (Figure 6E). Finally, when accounting for the same risk factors, HR of stroke per SD increase of EAT is 1.20 (95% CI: 1.08-1.32; *P =* 0.02) (Figure 6F). Results with statistically selected risk factor adjustment are shown in Supplemental Figure 4.

Adding EAT volume into a clinical model led to significant improvement in the ability to detect obstructive CAD on CCTA (any 1 coronary vessel with ≥50% stenosis on CCTA) cross-sectionally (ΔAUC: 0.103; *P <* 0.01) (Figure 6G) and improved the ability to predict future MI (both fatal and nonfatal) longitudinally (ΔAUC: 0.07; *P <* 0.01) (Figure 6H).

### A single predictive value of EAT volume for long-term disease risk

To generate distinct clinical groups based around a single EAT volume cutpoint, the SCOT-HEART trial population was dichotomized into high vs low EAT volume groups based on an optimum cutoff of 169.9 cm^3^ for the 3 primary outcomes of interest: all-cause mortality; fatal/nonfatal MI; and fatal/nonfatal stroke. This cutoff was derived from a weighted Youden J index analysis to generate a single value for application. Utilizing this cutpoint, there was significantly different adjusted HRs for the low EAT group (<169.9 cm^3^) vs the high EAT group (≥169.9 cm^3^) for all outcomes. All analyses included multivariable adjustment for CVD risk factors including BMI.

**Figure 7:**
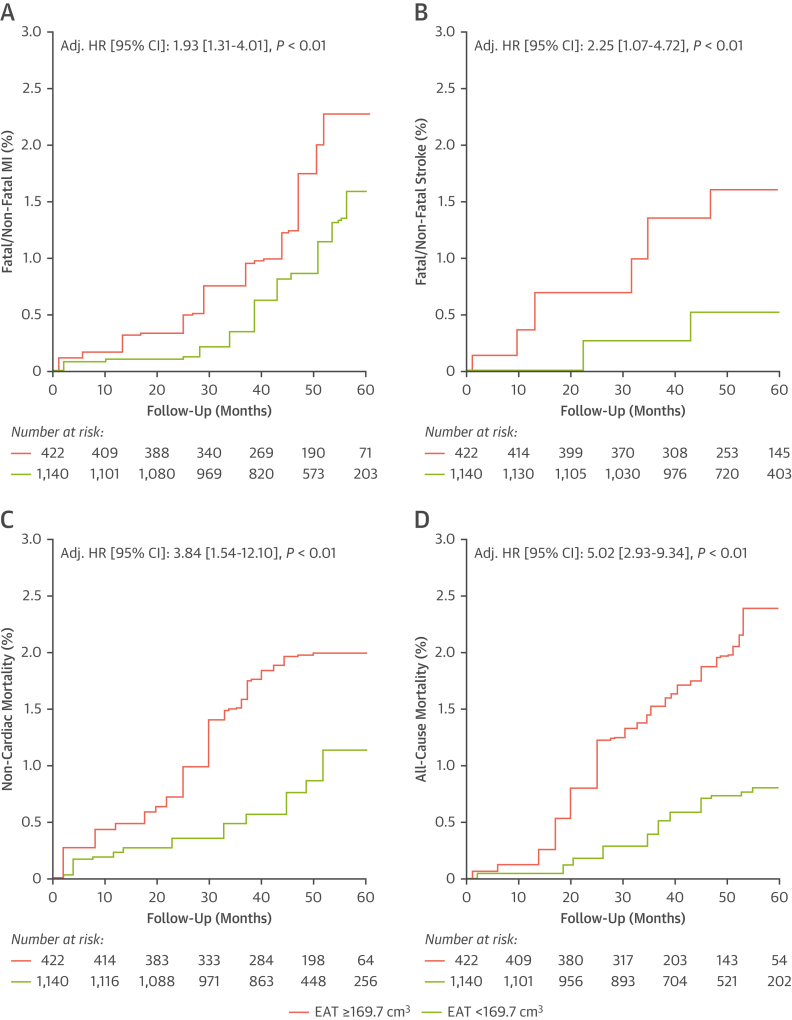
High EAT Volume Increases Risk of Major Adverse Events When Assessed With a Single Cutpoint Utilizing a single cutpoint for patients considered at high risk in SCOT-HEART (high risk = EAT ≥169.9 cm^3^), Kaplan-Meier curves for **(A)** fatal/nonfatal MI, **(B)** fatal/nonfatal stroke, **(C)** noncardiac mortality, and **(D)** all-cause mortality are demonstrated. All HRs are adjusted for age, sex, BMI, hypertension, diabetes mellitus, CAC score (log-transformed) and obstructive CAD as derived from CCTA. Abbreviations as in Figures 1, 2, 5, and 6.

High EAT volume values (≥169.9 cm^3^ vs <169.9 cm^3^) were associated with a higher prospective risk for both fatal and nonfatal MI (adjusted HR: 1.93; 95% CI: 1.31-4.01; *P <* 0·01) (Figure 7A), both fatal and nonfatal stroke (adjusted HR: 2.25; 95% CI: 1.07-4.72; *P <* 0·01) (Figure 7B), noncardiac mortality (adjusted HR: 3.84; 95% CI: 1.54-12.10; *P <* 0·01) (Figure 7C), and all-cause mortality (adjusted HR: 5.02; 95% CI: 2.93-9.34; *P <* 0·01) (Figure 7D).

### EAT volume and postoperative AF risk

Utilizing 250 scans from patients in the AdipoRedOx study, the longitudinal associations between EAT volume and in-patient postoperative AF (>30 seconds of AF on monitoring) and long-term AF (paroxysmal, persistent, or chronic) following surgery were investigated.

Again, a weighted Youden J index analysis was used to generate a single value to dichotomize the AdipoRedOx study population into high vs low EAT volume groups based on an optimum cutoff of 198.7 cm^3^ for the 2 primary outcomes of in-hospital postoperative AF and long-term postoperative AF. Utilizing this cutpoint, there was significantly different adjusted HRs for the low EAT group (<198.7 cm^3^) vs the high EAT group (≥198.7 cm^3^) for both outcomes.

**Figure 8:**
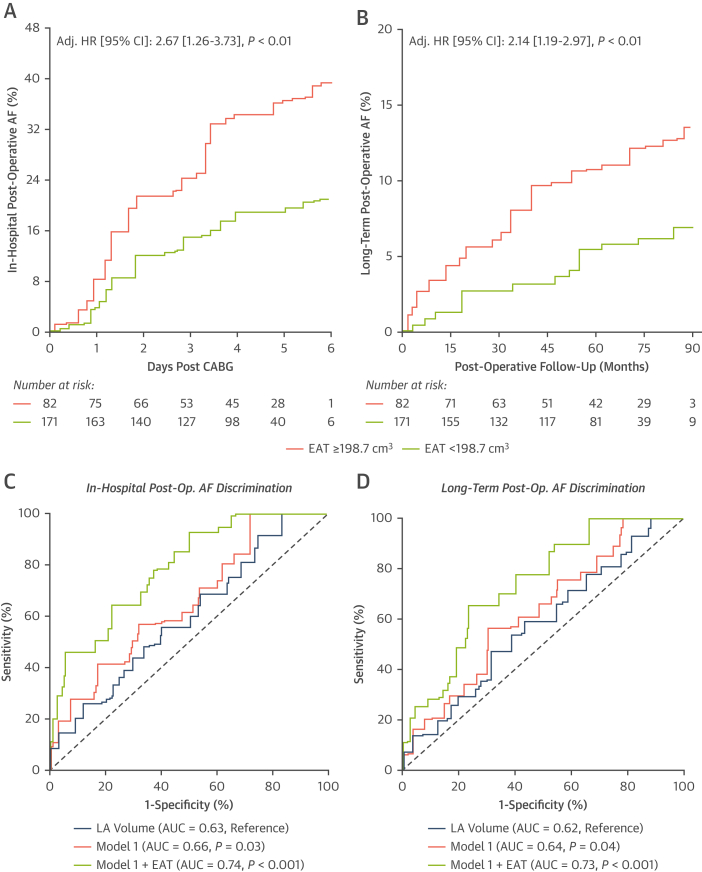
Prognostic Value of EAT Volume for Postoperative AF Kaplan-Meier curve and adjusted HR for in-hospital postoperative (post-op.) atrial fibrillation (AF) **(A)** and long-term postoperative AF **(B),** with sample dichotomized by Youden J index–derived cutpoint of EAT volume (high risk ≥198.7 cm^3^; low risk <198.7 cm^3^), expressed per SD increase of EAT volume. Adjustment is made for age, sex, hypertension, diabetes, CAC score, and BMI. **(C,D)** Time-dependent ROC curves for discrimination of in-hospital postoperative AF **(C)** and long-term postoperative AF **(D).** CCTA–derived left atrial (LA) volume **(blue)** is shown alone; model 1 **(red)** consists of age, sex, hypertension, diabetes, CAC score, and BMI. The addition of EAT volume into model 1 is demonstrated **(green)**. Abbreviations as in Figures 1, 2, 5, and 6.

There were 97 events of in-hospital postoperative AF (38.8%) and 48 cases of new-onset AF (19%) in nationwide NHS Digital data for the AdipoRedOx cohort. The Kaplan-Meier curve for in-hospital postoperative AF following cardiac surgery for high (≥198.7 cm^3^) vs low (<198.7 cm^3^) EAT volume. High EAT volumes were associated with a significantly greater risk for in-patient postoperative AF following adjustment for CVD risk factors, with HR of 2.67 (95% CI: 1.26-3.73; *P <* 0.01), per SD increase in EAT volume (Figure 8A). Equally, for long-term new-onset AF following cardiac surgery, high-risk EAT volumes were associated with a significantly greater risk for long-term AF, with HR of 2.14 (95% CI: 1.19-2.97; *P <* 0.01), per SD increase in EAT volume (Figure 8B).

The addition of EAT volume into a CVD risk factor model significantly improved the prediction of new-onset in-hospital AF in receiver-operating characteristic curve analysis (Figure 8C) with ΔAUC of +0.0.8 (*P =* 0.03) for CVD risk factor model 1 with the addition of EAT volume, and ΔAUC of +0.11 (*P <* 0.001) with the addition of the risk factor model plus EAT volume on top of CCTA-derived left atrial (LA) volume alone. The same was found for new-onset long-term AF (Figure 8D), with ΔAUC of +0.09 *(P =* 0.04) for risk factor model 1 with the addition of EAT volume, and ΔAUC of +0.11 (*P <* 0.001) over LA volume alone.

Identical analysis with adjustment for a selection of AF-specific risk factors, including LA volume and N-terminal pro–B-type natriuretic peptide, is presented as Supplemental Figure 6, with EAT volume retaining significance in all models. There was no significant change in results for all postoperative AF risk analysis when BMI was replaced with waist-hip ratio (Supplemental Figure 7).

## Discussion

In this study we developed a deep-learning model for automated segmentation and quantification of EAT from CCTA images. The model was then validated in multiple cohorts, including commonly occurring challenging populations where manual segmentation is extremely difficult because of artifacts with good performance. Then we applied this automated model to the SCOT-HEART cohort, demonstrating a good prognostic value of EAT volume for all-cause mortality and cardiovascular events, as a possible measure of unhealthy visceral obesity relevant to cardiometabolic dysfunction, regardless of whether EAT volume was used as a continuous variable or when used with a cutoff. Contrary to pericoronary Fat Attenuation Index, which captures the degree of coronary artery inflammation and is predictive of cardiac (but not of noncardiac) mortality, we now demonstrate that EAT volume is predictive of noncardiac mortality, confirming its role as a broader biomarker of visceral obesity that affects survival in a broader sense. We also demonstrate that this measurement has important prognostic value for postoperative AF in patients undergoing cardiac surgery, beyond known postoperative risk models including LA volume and N-terminal pro–B-type natriuretic peptide. Fully automated measurement of EAT volume incorporated into routine interpretation of CCTA promises to significantly improve the risk stratification of patients across several important clinical outcomes.

We developed a deep-learning model utilizing a single network for the fully automated and rapid quantification of EAT volume from CCTA. Previous automated models for EAT quantification have predominantly been in small cohorts.^12^^,^^13^ The most relevant model is by Commandeur et al^14^ who developed a convolutional neural network capable of automated EAT segmentation and tested in the EISNER (Early Identification of Subclinical Atherosclerosis by Noninvasive Imaging Research) cohort; however, no head-to-head comparison is made in this study. The model developed here achieves accurate EAT volume quantification in technically challenging yet commonly occurring clinical populations. The need for any artificial intelligence–based radiology approaches to be applicable for all-comers is fundamental to patient acceptance and the future uptake of such technology. The DLN reduced EAT quantification time from an average of 18 minutes when performed manually, to an average of 12 seconds, rendering this tool usable in the clinical environment without adding workload to clinical teams.

EAT volume has previously been found to be associated with CVD metrics and outcomes including the presence of atherosclerosis,^15^ as well as coronary calcification progression.^16^ EAT is a source of numerous proinflammatory mediators that circulate well beyond the microcirculation of the heart to exert paracrine and endocrine effects on the cardiovascular and endocrine systems.^5^ We found EAT volume to be a significant predictor of all-cause mortality even with the exclusion of cardiac deaths. This suggests that the EAT may play a clinically significant role in broader metabolic diseases beyond atherosclerotic CAD. We propose that EAT volume should be treated as the gold standard for the detection of metabolically unhealthy visceral obesity and could form part of routine clinical interpretation of CCTA. This would shift the focus of CCTA examination as a purely structural assessment of the coronary arteries toward a more universal assessment of cardiovascular risk that considers a key visceral, metabolically sensitive, tissue depot.

EAT is in continuous bidirectional communication with the cardiovascular system.^5^ When there is discordance between adipose tissue and the cardiovascular system the former is thought to shift function and exerts detrimental effects on the vessels and the heart muscle,^17^ which may predispose the patient to adverse outcomes such as those that we investigated. We found that EAT volume is predictive of nonfatal MI and stroke independent of BMI and following adjustment for other relevant disease risk factors.

We demonstrate that EAT volume is an independent predictor providing incremental value for postoperative AF regardless of patient BMI, LA volume, N-terminal pro–B-type natriuretic peptide, and other AF risk factors, indicating an important role for EAT in driving postoperative arrhythmogenesis. It is proposed that proinflammatory cytokines diffuse locally from dysfunctional EAT into the myocardium and contribute toward atrial myopathy,^18^ which drives the risk of AF.

### Study limitations

We did not have detailed adiposity data (eg, waist-hip ratio) or mortality data available within the SCOT-HEART trial population to investigate the exact causes of noncardiac mortality that could be driving our finding of elevated risk of all-cause mortality conveyed by EAT volume. There were only 4 cardiovascular deaths during the 5-year follow-up in the SCOT-HEART trial, limiting any cardiac mortality analysis. Other challenging clinical CCTA populations exist, such as those with congenital cardiac conditions, for which we lacked enough cases for testing of the deep-learning algorithm. Short-term AF risk data following cardiac surgery relied on inpatient monitoring only, without device monitoring following discharge.

## Conclusions

We present a new deep-learning model that allows accurate, reproducible, and rapid quantification of EAT volume on routine CCTA scans. We demonstrate that assessment of EAT volume can improve risk assessment for cardiovascular and noncardiovascular outcomes independently of other risk factors including BMI. EAT volume is predictive of all-cause mortality and noncardiac mortality, MI, and stroke. EAT volume also conveys significant independent risk of post–cardiac surgery AF. Incorporating automated EAT volume quantification in CCTA reading protocols could improve global cardiometabolic risk assessment and treatment planning for patients who undergo CCTA investigation, independently of the presence of coronary atherosclerosis.

Perspectives **COMPETENCY IN MEDICAL KNOWLEDGE:** The automated quantification of EAT volume through an artificial intelligence approach on routine CCTA scans may allow improvements in disease risk assessment for cardiovascular events such as MI and stroke.

**TRANSLATIONAL OUTLOOK:** The introduction of automated EAT assessment into standard CCTA interpretation could add significant value to patient care through enhanced prediction of disease risk for conditions such as CAD, stroke, and AF.

## Funding Support and Author Disclosures

This study received support from the British Heart Foundation (grant TG/19/2/34831) and the European Union Commission Horizon 2020 program via the Machine Learning Artificial Intelligence Early Detection Stroke Atrial Fibrillation (MAESTRIA) Consortium (grant 965286). Drs Siddique, Tomlins, and Shirodaria are employees of Caristo Diagnostics Ltd. Dr Williams has received support from the British Heart Foundation (grant FS/ICRF/20/26002); and has served on the Speakers Bureau for Canon Medical Systems. Dr Adlam has received support from the Leicester National Institute of Health Research Biomedical Research Centre; has received research funding and in-kind support for unrelated research from AstraZeneca Inc; has received an educational grant from Abbott Vascular Inc to support a clinical research fellow for unrelated research; and has also conducted consultancy for GE Inc to support research funds for unrelated research. Drs Shirodaria, Neubauer, Channon, and Antoniades are founders, shareholders, and directors of Caristo Diagnostics Ltd, a CT-image analysis company. Dr Antoniades has received support from the British Heart Foundation (grants CH/F/21/90009, TG/19/2/34831, and RG/F/21/110040), Innovate UK (grant 104472), and the National Consortium of Intelligent Medical Imaging through the Industry Strategy Challenge Fund (Innovate UK grant 104688); and is also the inventor of patents US10,695,023B2, PCT/GB2017/053262, GB2018/1818049.7, GR20180100490, and GR20180100510, which are licensed through exclusive license to Caristo Diagnostics. All other authors have reported that they have no relationships relevant to the contents of this paper to disclose.
