# Supplementary material for: Deep-Learning for Epicardial Adipose Tissue Assessment With Computed Tomography: Implications for Cardiovascular Risk Prediction
Source: JACC Cardiovasc Imaging. 2023 Jun;16(6):800–16. doi: 10.1016/j.jcmg.2022.11.018 (PMC10663979; doi:10.1016/j.jcmg.2022.11.018)
Supplement: Supplemental Material [file mmc1.docx]

**Supplemental Methods**

Study ethics

All studies utilized in this manuscript complied with the human research ethics committees of the coordinating institute. The ORFAN Study (15/SC/0545) and the AdipoRedOx Study (C11/SC/0140) are sponsored by the University of Oxford and are both conducted with approval of the South Central – Oxford C Research Ethics Committee, as part of the Oxford Heart Vessels and Fat (ox-HVF) program. The SCOT-HEART trial was sponsored by the University of Edinburgh and was conducted with the approval of the South-East Scotland Research Ethics Committee (NCT01149590). ORFAN USA sites research protocol was approved by all local institutional review boards, including material and data sharing agreements, with waiver of individual informed consent.

Study populations

ORFAN study

The ORFAN study is an Ox-HVF study that enrolls adult patients who receive clinically indicated CCTA for all accepted indications at collaborating hospitals. Enrolment is through individual clinical sites and includes 13 NHS Trusts in England and 2 clinical sites in the USA. ORFAN scans utilized in this study were performed between January 2015 through December 2020. For each patient included in the study the CCTA scan is pseudo-anonymized and delivered to The Oxford Academic Cardiovascular CT (OXACCT) Core Lab for analysis. A total of 2,200 ORFAN patients’ scans were utilized for the manual training of the deep-learning model while 600 further unseen scans were utilized for feedback learning (where the scan is fed into the model for automated segmentation, the output segmentation is then corrected by a human expert before being fed back into the model). A total of 100 unseen UK ORFAN scans were utilized for preliminary evaluation of the model, and 200 unseen UK ORFAN scans were used for internal validation of the model. No outcome analysis of the ORFAN cohort was performed. A total of 720 ORFAN USA scans (Cleveland Clinic, OH) were used only for external validation of the model and remained sequestered throughout the development and internal validation process.

AdipoRedOx study

The AdipoRedOx study is an Ox-HVF study that enrolls cardiac surgery patients at the Oxford University Hospitals NHS Foundation Trust, UK. The study is designed to investigate the role of inflammation and redox-state biology on heart disease through the collection of heart, vessel, and adipose tissue at the time of surgery. Relevant to this study, participants also undergo a CCTA within 6 weeks following surgery. Scans and relevant patient data from 253 participants were utilized in this study for the validation of the automated EAT quantification tool in challenging post–cardiac surgery patients.

SCOT-HEART trial

SCOT-HEART is an open-label, randomized, controlled, parallel-group trial that was performed at 12 centers across Scotland, commencing in 2009. Adult patients (n = 4,126) referred to outpatient cardiology clinics for stable chest pain were randomly assigned in a 1:1 ratio to standard care plus CCTA or to standard care alone. No standard-care patients were utilized in this study. The trial has been described in detail previously.^1,2^ The median duration of follow-up was 4.8 years. In this study, 1,558 eligible patients who had been randomized to the CCTA arm of the study were utilized.

Risk factors

In regression models, hypertension was defined based on the presence of a documented diagnosis or treatment with an antihypertensive regimen, according to the relevant clinical guidelines.^3^ Similar criteria were applied for the definition of hypercholesterolemia and diabetes mellitus.^4,5^ Valve disease was defined as the presence of any documented aortic or mitral valve stenosis or regurgitation (of any severity), or previous valve repair and/or replacement procedure, including minimal invasive methods. Previous heart surgery was defined as any previous documented coronary artery bypass graft procedure or valve replacement or repair procedure (not including TAVR). Active smoking was defined as any documented active smoking of any amount of tobacco products, including all tobacco products. All SCOT-HEART baseline variables have been described in further detail elsewhere.^6^

Outcomes

SCOT-HEART study

The cross-sectional outcome of AF was defined as any documented diagnosis of paroxysmal, persistent, or chronic AF within a patient’s medical history, including self-report of the patient at the time of enrolment in the study. Outcomes within SCOT- HEART trial are all defined within the study protocol.^6,7^ Importantly, MI was defined as per the Universal Definition of the Joint ESC/ACCF/AHA/WHF Task Force for the Redefinition of Myocardial Infarction.^8^ For the purposes of this study, the investigators followed the original SCOT-HEART investigators adjudication of events. This adjudication occurred according to the guidelines of the ACC/AHA^9^ and the Academic Research Consortium for definition of the cause of death.^10^ Cardiac mortality within the study was defined as “any death caused by proximate cardiac causes (eg, myocardial infarction, low-output heart failure, fatal arrhythmia).”^6^ Sudden cardiac death was also included within this group.^9,10^ Any other death that was not captured by the previous definition was classified as of noncardiac cause. Noncardiac deaths included vascular causes such as stroke and embolism within the pulmonary circulation. Deaths were classified as “deaths of unknown cause” only when information on the exact cause could not be obtained with certainty, which was at the discretion of the local SCOT-HEART investigators. The protocol of the SCOT-HEART study required that all adjudication of study events and cause of death be performed at each local site by the local study investigators. These investigators were not involved in the CT scan analysis or the statistical analysis of the study data. Events were ascertained via patient chart review, death certificate review and/or telephone follow-up or in-person verification with family members. No patient events were readjudicated for this study. All information utilized in this study was sent for statistical analysis to an independent team based at the University of Oxford, United Kingdom, which performed all analyses blind to scan data.

AdipoRedOx study

Immediate postoperative AF outcomes were collected in hospital during the in-patient stay following the surgery. AF in this context was defined as >30 seconds of AF captured on continuous electrocardiographic monitoring. Patients were on telemetry monitoring of heart rhythm throughout their in-patient stay. If telemetry was ceased by the treating team, the patient was censored at that time in analysis.

The long-term post-operative AF outcome was collected from NHS Digital data via linkage with study participant identifiers (NHS number and date of birth). Linkage was made to hospital episode statistics data for all regular admissions, outpatient episodes, emergency presentations and critical care episodes following the surgery. The long-term AF outcome is a merger of relevant ICD-10 codes for AF including codes that capture the diagnosis of paroxysmal AF, persistent AF, chronic AF, and AF unspecified (codes I48.0, I48.1, I48.2, I48.9, respectively). If any of these codes were entered for a patient over the follow-up period, this was counted as an event.

Manual CCTA segmentation

All analyses were performed using the standard operating procedures of the Oxford Academic Cardiovascular CT (OXACCT) core lab at the Acute Vascular Imaging Centre (AVIC) in the University of Oxford. Manual segmentation of the whole heart and the pericardium was performed on randomly selected ORFAN CCTA scans for the training of the deep-learning model. The manual segmentation was undertaken by 2 independent readers. Both readers are trained members of the OXACCT Core Lab and have undertaken training in the specific whole heart segmentation technique required. The training of the readers was overseen by the Director of the Core Lab (SCCT level 3 qualification). According to the Quality Management System of the OXACCT core lab, the readers receive training based on a well-defined and controlled SOP. Before qualification as expert readers, the core lab members need to achieve <5% variability in manual segmentation of the pericardium compared to the gold standard segmentations included into the training and testing package, and they perform at least 150 supervised manual segmentations with assessment and peer-review prior to commencing any research analysis. The standard operating procedures of the Core Lab mandate the use of quality control assessment of segmentations performed for both research and industry.

The whole heart within the pericardium was segmented between the bifurcation of the pulmonary trunk superiorly and the apex of the pericardium inferiorly. CaRi-Heart^®^ version 2.2.1 (Caristo Diagnostics Ltd, Oxford, UK) was utilized for manual imaging segmentation (see Supplemental Figure 1 for an example of manual segmentation). All voxels within the pericardium including the visceral pericardium were included. Examination of EAT volume segments for intraobserver bias was excellent with both expert human readers having excellent intraclass correlation coefficient in a test of 20 blinded repeat reads of the same case (reader 1, ICC [95% CI] = 0.987 [0.980-0.999] *P <* 0.001; reader 2, 0.984 [0.976-0.998] *P <* 0.001; interval between reads 2 weeks). Following initial training with 2,200 manually segmented CCTA, the model was further enhanced by 3 iterations of feedback learning performed on unseen CCTA scans from the ORFAN study. These unseen scans were fed into the model in batches of 200, then the output segmentations were manually corrected by 2 experts readers (same readers as the training analysis) before being fed back into the deep-learning model.

The extraction of EAT volume from the heart segmentation

The extraction of EAT volumes from the segmentation of the whole heart was achieved by automated selection of adipose tissue voxels. The Hounsfield unit (HU) range of −190 HU through to −30 HU was utilized, adhering to the conventional definition of adipose tissue density on CCTA.^11-13^ The extraction of the EAT volume on a per-voxel basis from within the whole heart segment was fully automated in a bespoke module within the CaRi-Heart^®^ platform, created especially for the purpose of the quantification of EAT from CT segmentations. An example of EAT segmentation is shown in Figure 3B, with manual segmentations and automated segmentation shown independently, and merged.

Traditional CCTA interpretation methods

CAC score

In the SCOT-HEART trial population coronary artery calcium score was quantified by the Agatston method on noncontrast cardiac CT scans using commercially available software (Aquarius Workstation® V.4.4.11-13, TeraRecon Inc, Foster City, CA, USA), in those patients with an indication for CCS assessment.

Obstructive CAD

Included SCOT-HEART trial scans has been previously reviewed by 2 independent clinical researchers within the OXACCT Core Lab, as published previously.^14^ Obstructive CAD was defined as the presence of at least 1 coronary stenosis ≥50% on CCTA.

Coronary computed tomography angiography technical parameters

ORFAN study

Participants in the ORFAN study in the UK underwent CCTA scans at NHS Trust hospitals including Oxford University Hospital NHS Foundation Trust, University Hospitals of Leicester NHS Trust, Royal United Hospitals Bath NHS Foundation Trust, Milton Keynes University Hospital NHS Foundation Trust. All scans were performed according to local clinical procedures, with minimal variance between sites. Heart rate was optimized using intravenous injection of beta-blockers and sublingual glyceryl-trinitrate (800 μg) was also administered to achieve maximum coronary vasodilatation if needed. CCTA was performed following intravenous injection of 50-70 mL of iodine based contrast medium (typically Niopam 370, BRACCO UK Ltd) at a flow rate of 5.5-6.5 mL/sec (axial slice thickness on Canon scanners was typically 0.5 mm, with Siemens typically between 0.65 mm and 0.75 mm, rotation time was 0.35 seconds, detector coverage was up to 160 mm, and tube energy was selected based on body habitus and according to local clinical practice). Prospective image acquisition was used by ECG-gating at 75% of cardiac cycle (with 100 msec padding if required). If not possible, retrospective image acquisition was used (eg, in the presence of irregular rhythm). For ORFAN USA sites, a total of 2,246 scans were available for inclusion. A random selection of 720 were selected for inclusion in the cohort. The majority of the CCTA scans (n = 627, 87·0% of the total number of scans) were performed in a 256-slice Brilliance iCT scanner (Philips Medical Systems, Best, Netherlands), with the remainder using a 2 × 128-slice Definition Flash scanner (Siemens Healthcare, Erlangen, Germany) (n = 70, 9.7%) and a 2 × 192-slice Somatom Force CT scanner (Siemens Healthcare, Forchheim, Germany) (n = 23, 3·2%). In patients with heart rate >60 beats/min, 5 mg of intravenous metoprolol (with incremental 5 mg doses up to a maximum dose of 30 mg) or intravenous diltiazem (5 mg increments up to 20 mg maximum), if the heart rate remained above 60 beats/min once the patient was positioned on the CT table. Patients also received 0·3 mg of nitroglycerin sublingually immediately before CCTA and iodinated contrast (Omnipaque 350, General Electric, Milwaukee, USA) was administered at flow rate of 5-6 mL/sec.

For all ORFAN sites, scans with profoundly poor-quality images, more than 2 missing slices, severe beam hardening or significant step artifacts were excluded from the analysis. Minor to moderate scan artifacts were acceptable and included in analysis.

AdipoRedOx study

Participants in AdipoRedOx underwent CCTA using a 64-slice scanner (LightSpeed VCT, General Electric), as previously described.^15^ Heart rate was optimized using intravenous injection of beta-blockers and sublingual glyceryl-trinitrate (800 μg) was also administered to achieve maximum coronary vasodilatation. A noncontrast prospectively ECG triggered axial acquisition CT scan was obtained (0.35 seconds rotation time, 2.5 mm axial slice thickness, 20 mm detector coverage, tube energy of 120 kV and 200 mA) with the carina and the diaphragm used as cranial and caudal landmarks, respectively. Lung field of view was extended to cover the entire thoracic soft tissue (for subcutaneous adipose tissue analysis). CCTA was performed following intravenous injection of 95 mL of iodine based contrast medium (Niopam 370, BRACCO) at a flow rate of 4.5-6 mL/sec (tube energy of 120 kVp, axial slice thickness of 0.625 mm, rotation time of 0.35 seconds, detector coverage of 40 mm). Prospective image acquisition was used by ECG-gating at 75% of cardiac cycle (with 100 msec padding for optimal imaging of the right coronary artery if required). There were no exclusions from the AdipoRedOx cohort based on scan parameters.

SCOT-HEART trial

CCTA scans were performed using either 2 64 detector row scanners (Brilliance 64, Philips Medical Systems, Netherlands, and Biograph mCT, Siemens, Germany) or a 320 detector row scanner (Aquilion ONE, Toshiba Medical Systems, Japan) at 3 imaging sites according to each site’s local protocol.^1,2^ A total of 1,786 patients were available for analysis. Scans performed at tube voltage settings other than 100 or 120 kVp, and scans with profoundly poor-quality images (unsuitable for human or machine analysis), 2 or more missing slices, severe beam hardening, or significant step artifacts were excluded from the analysis (n = 228), resulting in a study dataset of 1,558 CCTA scans.

Scan retrieval

All CCTAs from each study were first anonymized locally, or in the cloud at the time of transfer, and sent to the Oxford Academic Cardiovascular Computed Tomography (OXACCT) core lab (Oxford, UK) for either manual analysis on dedicated workstations or for sequestering for later automated validation analysis.

The training of the deep-learning model for automated segmentation of the whole heart within the pericardium

The 2,200 manually whole heart segmented CCTA scans were individually normalized to have 0 mean and unary SD, as on input networks performed better in this intensity range. A convolutional neural network was used for segmentation by casting them to voxel-wise classification. The network was trained to predict whether the central voxel of a patch is or is not within the bounds of the pericardium depending on the content of the surrounding 3D patch.

A fully automatic method was employed using 3D Residual-U-Net neural network architecture [3] for 3D volumetric segmentation of CCTA data. A building block of a Residual-U-Net is called a residual block or skip connection. The activation of a layer is fast-forwarded to a deeper layer in the neural network in a residual block. This allows training much deeper neural networks without the issue of vanishing gradients. The architecture resembles auto encoder framework and consist of 4 down- sampling and 4 up-sampling blocks which are connected by a bridge block. Feature size is halved after passing through every down-sampling block by a max-pooling layer at its base. At the beginning of every up-sampling block the feature size is doubled using transposed convolution layer with stride of size 2 for each dimension. Passing information through interconnections was achieved by using 0 padding. After every convolution operation we fill the newly computed vector with 0s to its original length and therefore both convolution layers use the same size of the input vector. During training, the parameters of the kernels were optimized using Adam optimizer, with the target of minimizing the error between the predictions and the true labels. The hyper-parameters of the network were tuned following the first and second stages of the feedback learning process, that is after he first and second cycles of scan (n = 200) input and correction.

The CNN as shown in Figure 3A of the main text demonstrates the layers of model. The light green boxes (Conv+RelU) represent a convolutional layer. These layers capture the spatial relationship between CCTA voxels to receive image features using small patches of input data by learning local connectivity patterns followed by RelU activation to ensure nonlinearity, sparsity, and a reduced likelihood of vanishing gradient. Yellow boxes (Concat) represents residual interconnections to allow gradients to pass through the network directly by skipping over series of convolution and nonlinear activation functions to solve the degradation problem of the deep networks, and concatenation to add tensors of both connections. Max pooling layer is represented by dark green color box (MaxPool) to extract sharp and smooth features. Orange boxes (UnPool) represents the up-sampling step to revert the effect of the max pooling operation. The purple box (Sigmoid) represents the output layer using sigmoid function. This outputs values in the range 0-1 representing the probability of a voxel being within the desired segmentation of the heart pericardium. The gray horizontal lines represent concatenation of decoder paths with the corresponding feature map from the encoder path.

The network architecture was trained using Keras framework using Tensorflow 2.2 (Google Brain, Mountain View, CA, USA) as backend. The network used a binary cross-entropy as a loss function and Dice coefficient as performance metric.

Automated left atrium segmentation

Left atrium volume was utilized as a covariate in analysis of the relationship between EAT volume and postoperative AF risk within the AdipoRedOx study. In the AdipoRedOx study, LA volume was calculated via automated quantification upon the same CCTA utilized for EAT quantification. The automated segmentation was trained in the same manner to the whole heart segmentation—via a Residual-U-Net neural network architecture for 3D volumetric segmentation the LA chamber, including the LAA. The training cohort for this analysis was the same as described for the whole heart in this manuscript—the UK ORFAN study population. The internal validation cohort was 200 unseen UK ORFAN scans, with resulting concordance correlation coefficient (CCC) for human versus machine of 0.981. The external validation cohort was in 720 USA ORFAN CCTAs, with a CCC of 0.971. No AdipoRedOx study scans were utilized in the training or validation of the LA volume quantification tool. The automated tool was incorporated into the CaRi-Heart analysis platform (Caristo Diagnostics Ltd, Oxford, UK).

Preliminary internal validation of the whole heart segmentation DLN

EAT was segmented manually in 100 randomly selected scans from the UK arm of ORFAN, all previously sequestered from the algorithm, prior to the same set of scans being segmented by the automated deep-learning algorithm. This analysis occurred prior to the initiation of the feedback learning cycles and represents a preliminary validation of the model (Supplemental Figure 2). CCC was excellent between human expert and the machine at 0.960, and the bias was nonsignificant at 5.5 [−8.0 to 14.7] cm^3^; *P =* 0.22.

For the whole heart volume in the same 100 scans there was minimal variability between a single human expert and machine (Supplemental Figure 3). CCC was excellent between human expert and the machine at 0.969, and the bias was nonsignificant at 29.2 [−30.3 to 77.5] cm^3^; *P =* 0.18.

Quantification of blood markers

AdipoRedOx

NT-proBNP was calculated from blood samples collected in an EDTA tube intraoperatively for each participant. Plasma BNP was quantified by chemiluminescent-microparticle immunoassay (Architect BNP, Abbott, Germany).

SCOT-HEART trial

Non-HDL cholesterol was calculated along with a complete lipid panel as previously described.^6,7^

Supplemental statistical analysis

Participant demographics are summarized as numbers (percentages) or median (25th to 75th percentile) for categorical and continuous variables, respectively. For intraobserver repeatability, intraclass correlation coefficient was calculated with 20 repeat cases per reader.

Whenever utilized, coronary artery calcium (CAC) score was log-transformed before inclusion in regression models (ln[CAC + 1]).

For cross-sectional analysis of disease risk conveyed by EAT volume, multiple-adjusted logistic regression was used for calculation of the odds-ratio of prevalent disease (AF at time of CCTA and obstructive CAD from CCTA) at the time of the CCTA given increase in EAT by 1 SD. Within the main text, results are presented with adjustment for all models with a uniform set of covariates. Supplemental results are presented with a statistical approach to risk factor adjustment. Longitudinal assessment of the prognostic value of EAT volume was performed by multivariable Cox regression. Both odds ratios and HRs are reported per SD increase in EAT volume. The variables that are included in each supplemental model of the SCOT-HEART trial analysis are the risk factors that have shown a statistically meaningful association with the relevant outcome (dependent variable) in univariate analysis, at the level of *P ≤* 0.1 (see Supplemental Table 1).

When calculating Youden’s J Statistic within the SCOT-HEART cohort, we weighted the influence of each outcome (all-cause mortality, fatal/nonfatal MI, and fatal/nonfatal stroke) according to the number of events in the population and then tested the prognostic value of the high versus low EAT volume (as a dichotomous variable) by multivariable Cox regression analysis after adjustment for relevant disease risk factors as listed in the results.

For analysis of risk of postoperative AF in CABG patients from the AdipoRedOx study, secondary analysis with AF specific risk factors was performed (see Supplemental Figure 6). The risk factors selected in this analysis were selected for their known relationship with new onset AF in the postoperative population following literature review.

Statistical analyses were performed predominantly in STATA SE version 15 (Stata Corp, College Station, TX, USA), with some analysis in the R environment (R for Windows 4.0.4). All tests were 2-sided and α was set at 0.05, unless specified otherwise.

**Supplemental Results**

Preliminary internal validation of the automated whole heart and EAT volume detection model vs human segmentation

EAT was segmented manually in 100 randomly selected scans from the UK arm of ORFAN, all previously sequestered from the algorithm, prior to the same set of scans being segmented by the automated deep-learning algorithm. This analysis occurred prior to the initiation of the feedback learning cycles and represents a preliminary validation of the model (Supplemental Figure 1). CCC was excellent between human expert and the machine at 0.960, and the bias was nonsignificant at 5.5 [−8.0 to 14.7] cm^3^; *P =* 0.22.

For the whole heart volume in the same 100 scans there was minimal variability between a single human expert and machine (Supplemental Figure 2). CCC was excellent between human expert and the machine at 0.969, and the bias was nonsignificant at 29.2 [−30.3 to 77.5] cm^3^; *P =* 0.18.

Cross-sectional clinical correlations with statistical approach to co-variate selection

At the time of the CCTA, application of the fully automated segmentation tool for quantification of EAT volume was found to be a significant independent predictor of the presence of AF at time of CCTA and obstructive CAD from CCTA (any 1 coronary vessel with ≥50% stenosis on CCTA), within 1,558 patients randomized to receive CCTA in the SCOT-HEART trial population. When accounting for AF risk factors the odds ratio (OR [95% CI]) of AF at time of CCTA per SD increase of EAT was 1.20 [1.06-1.45] *P =* 0.03 (Supplemental Figure 4A). When accounting for CAD risk factors the OR of obstructive CAD from the CCTA per SD increase of EAT was 1.12 [1.03-1.30] *P =* 0.01 (Supplementary Figure 4B).

Longitudinal EAT volume clinical correlations with statistical approach to covariate selection

Median follow-up for the 1,558 patients randomized to receive CCTA in SCOT-HEART which were analyzed was 4.8 years. There were 35 deaths of all causes (2.25%) of which 4 (0.25%) were deaths related with coronary heart disease. There were 8 fatal/nonfatal strokes (0.51%) and 39 fatal/nonfatal myocardial infarctions (2.5%).

The HR [95% CI] of all-cause mortality per SD increase of EAT was 1.24 [1.08-1.34] *P =* 0.03, after accounting for relevant general disease risk factors (Figure 6C). When adjusted for the same risk factors as Figure 6A, the HR [95% CI] of noncardiac mortality per SD increase of EAT volume was 1.14 [1.02-1.37] *P =* 0.04 (Supplemental Figure 4D). This constitutes a ∆HR of −0.10, confirming that EAT is a measure of visceral adipose tissue related with multiple fatal pathologies, unrelated to CAD. When accounting for CAD risk factors the HR of fatal/nonfatal MI per SD increase of EAT was 1.25 [1.08-1.45] *P =* 0.001 (Supplemental Figure 4E). Finally, when accounting for stroke risk factors the HR [95% CI] of fatal/nonfatal stroke per SD increase of EAT is 1.12 [1.03-1.27] *P =* 0.02 (Supplemental Figure 4F).

EAT volume and postoperative AF risk with AF specific risk factors

As is reported in the main manuscript, utilizing 250 scans from patients in the AdipoRedOx study, the longitudinal associations between EAT volume and in-patient postoperative AF and long-term AF following surgery were investigated.

Utilizing the same methods described in the main text, secondary analysis was performed with multivariable adjustment for relevant AF risk factors including LA volume and NT-proBNP (see Supplemental methods for quantification details). High EAT volumes were associated with a significantly greater risk for in-patient postoperative AF following adjustment for typical AF risk factors, with HR [95% CI] of 1.54 [1.12-3.33] *P <* 0.01, per SD increase in EAT volume (Supplemental Figure 6A). Equally, for long-term new-onset AF following cardiac surgery high risk EAT volumes were associated with a significantly greater risk for long-term AF following adjustment for typical AF risk factors, with HR [95% CI] of 1.34 [1.10-2.87] *P <* 0.01, per SD increase in EAT volume (Supplemental Figure 6B).

The addition of EAT volume into a clinical risk factor model, significantly improved the prediction of new-onset in-hospital AF in ROC curve analysis (Supplemental Figure 6C) with ΔAUC of +0.108 (*P <* 0.01) for risk factor model 1 with the addition of EAT volume, and ΔAUC of +0.221 (*P <* 0.001) with the addition of the risk factor model plus EAT volume on top of CCTA-derived LA volume alone. The same was found for new-onset long-term AF (Supplemental Figure 6D), with ΔAUC of +0.09 (*P <* 0.001) for risk factor model 1 with the addition of EAT volume, and ΔAUC of +0.141 (*P <* 0.001) over LA volume alone.

EAT volume and postoperative atrial fibrillation risk with waist-hip ratio included in analysis

Replacement of BMI with waist-hip ratio (WHR) in the original analysis as presented in Figure 8 did not alter the statistical significance of the results (Supplemental Figure 7). High EAT volumes were associated with a significantly greater risk for in- patient post-operative AF following adjustment for CVD risk factors including WHR, with HR [95% CI] of 12.57 [1.23-3.63] *P <* 0.01, per SD increase in EAT volume (Supplemental Figure 7A). Equally, for long-term new-onset AF following cardiac surgery high risk EAT volumes were associated with a significantly greater risk for long-term AF following adjustment for CVD risk factors including WHR, with HR [95% CI] of 2.16 [1.11-3.21] *P <* 0.01, per SD increase in EAT volume (Supplemental Figure 7B). The addition of EAT volume into a clinical risk factor model including WHR, significantly improved the prediction of new-onset in-hospital AF in ROC curve analysis (Supplemental Figure 7C) with ΔAUC of +0.05 (*P <* 0.01) for risk factor model 1 with the addition of EAT volume, and ΔAUC of +0.09 (*P <* 0.001) with the addition of the risk factor model plus EAT volume on top of CCTA derived LA volume alone. The same was found for new-onset long-term AF (Supplemental Figure 7D), with ΔAUC of +0.07 (*P <* 0.01) for risk factor model 1 with the addition of EAT volume, and ΔAUC of +0.1 (*P <* 0.001) over LA volume alone.

| **Supplemental Table 1. Standardized univariate analysis of variables for inclusion in multivariable models in addition to EAT volume** | | | |
| --- | --- | --- | --- |
| ***Variables*** | | ***Beta*** | ***P-***value******* |
| **Dependent variable: Presence of atrial fibrillation at the time of the CCTA** | | | |
| *Factors (independent variables) qualified for inclusion in the models* | | | |
|  | Age | 0.32 | 0.002 |
|  | Male sex | 0.19 | 0.005 |
|  | BMI | 0.16 | 0.01 |
|  | Hypertension | 0.15 | 0.009 |
|  | Diabetes | 0.09 | 0.05 |
|  | Valve disease | 0.10 | 0.09 |
|  | Previous heart surgery | 0.06 | 0.07 |
| *Factors (independent variables) tested but not qualified for inclusion in the models* | | | |
|  | Non-HDL cholesterol | -0.02 | 0.34 |
|  | Active smoker | 0.03 | 0.20 |
|  | Previous smoking history | -0.02 | 0.55 |
|  | Obstructive disease from CCTA | 0.19 | 0.21 |
|  | CAC Score | 0.09 | 0.49 |
| **Dependent variable: Obstructive CAD from CCTA (cross-sectional)** | | | |
| *Factors (independent variables) qualified for inclusion in the models* | | | |
|  | Age | 0.27 | 0.002 |
|  | Male sex | 0.17 | 0.005 |
|  | BMI | 0.19 | 0.009 |
|  | Hypertension | 0.17 | 0.01 |
|  | CAC score | 0.18 | 0.01 |
|  | Non-HDL cholesterol | 0.10 | 0.02 |
|  | Active smoker | 0.07 | 0.04 |
| *Factors (independent variables) tested but not qualified for inclusion in the models* | | | |
|  | Diabetes | 0.04 | 0.22 |
|  | Valve disease | 0.01 | 0.53 |
|  | Previous cardiac surgery | 0.04 | 0.29 |
|  | Previous smoking history | 0.09 | 0.19 |
| **Dependent variable: All-cause mortality** | | | |
| *Factors (independent variables) qualified for inclusion in the models* | | | |
|  | Age | 0.31 | 0.002 |
|  | Male sex | 0.19 | 0.01 |
|  | BMI | 0.19 | 0.04 |
|  | Hypertension | 0.09 | 0.05 |
|  | Diabetes | 0.10 | 0.09 |
|  | Active smoker | 0.09 | 0.06 |
| *Factors (independent variables) tested but not qualified for inclusion in the models* | | | |
|  | Non-HDL cholesterol | 0.08 | 0.22 |
|  | CAC score | -0.05 | 0.38 |
|  | Obstructive disease from CCTA | 0.08 | 0.25 |
|  | Valve disease | 0.04 | 0.34 |
|  | Previous cardiac surgery | 0.009 | 0.58 |
|  | Previous smoking history | 0.11 | 0.15 |
| **Dependent variable: Non-cardiac mortality** | | | |
| *Factors (independent variables) qualified for inclusion in the models* | | | |
|  | Age | 0.36 | 0.001 |
|  | Male sex | 0.12 | 0.01 |
|  | BMI | 0.09 | 0.04 |
|  | Hypertension | 0.03 | 0.09 |
|  | Diabetes | 0.13 | 0.07 |
| *Factors (independent variables) tested but not qualified for inclusion in the models* | | | |
|  | Non-HDL cholesterol | 0.03 | 0.28 |
|  | CAC score | -0.11 | 0.19 |
|  | Obstructive disease from CCTA | 0.03 | 0.22 |
|  | Valve disease | 0.10 | 0.33 |
|  | Previous cardiac surgery | 0.04 | 0.48 |
|  | Active smoker | 0.11 | 0.15 |
|  | Previous smoking history | 0.09 | 0.18 |

| **Dependent variable: MI** | | | |
| --- | --- | --- | --- |
| *Factors (independent variables) qualified for inclusion in the models* | | | |
|  | Age | 0.31 | 0.008 |
|  | Male sex | 0.09 | 0.09 |
|  | BMI | 0.15 | 0.02 |
|  | Hypertension | 0.11 | 0.05 |
|  | Non-HDL cholesterol | 0.09 | 0.08 |
|  | CAC score | 0.13 | 0.09 |
|  | Obstructive disease from CCTA | 0.16 | 0.04 |
| *Factors (independent variables) tested but not qualified for inclusion in the models* | | | |
|  | Diabetes | 0.06 | 0.08 |
|  | Valve disease | 0.03 | 0.45 |
|  | Previous cardiac surgery | -0.05 | 0.34 |
|  | Active smoker | 0.12 | 0.15 |
|  | Previous smoking history | 0.009 | 0.22 |
| **Dependent variable: Stroke** | | | |
| *Factors (independent variables) qualified for inclusion in the models* | | | |
|  | Age | 0.30 | 0.009 |
|  | Male sex | 0.12 | 0.02 |
|  | BMI | 0.16 | 0.01 |
|  | Hypertension | 0.09 | 0.02 |
|  | Valve disease | 0.07 | 0.08 |
|  | Previous heart surgery | 0.16 | 0.06 |
| *Factors (independent variables) tested but not qualified for inclusion in the models* | | | |
|  | Diabetes | 0.07 | 0.12 |
|  | Non-HDL cholesterol | 0.003 | 0.66 |
|  | Active smoker | 0.12 | 0.13 |
|  | Previous smoking history | 0.02 | 0.46 |
|  | Obstructive disease from CCTA | 0.006 | 0.59 |
|  | CAC score | 0.04 | 0.49 |
| *Significance set at *P ≤* 0.1  AF: atrial fibrillation; BMI: body mass index; CAC: coronary artery calcium score; HDL: high-density lipoprotein; MI: myocardial infarction | | | |

| **Supplemental Table 2. Outcomes from the prospective clinical cohorts** | | |
| --- | --- | --- |
| **Prospective follow-up** |  | |
|  | **AdipoRedOx**  ***cohort of cardiac surgery*** | **SCOT-HEART *outcomes cohort*** |
| Duration in-hospital (days) | 6 days (4.1-7.5) | not applicable |
| Duration long-term follow-up (months) | 5.6 years (3.8-6.5) | 4.8 years (4.2-5.7) |
| All-cause mortality | not applicable | 35 (2.25%) |
| Cardiac mortality | not applicable | 4 (0.26%) |
| Myocardial infarction* | not applicable | 39 (2.5%) |
| Stroke* | not applicable | 8 (0.51%) |
| Post-operative AF in hospital | 97 (38.3%) | not applicable |
| Post-operative AF long-term | 48 (19%) | not applicable |
| Data are median (IQR) or mean (%).  *Fatal and nonfatal.  AF: atrial fibrillation; MI: myocardial infarction | | |

**ORFAN Study Investigators**

**Chief Investigator:** Professor Charalambos Antoniades^1^

Investigators:

Dr Henry West^1^

Dr Alexios Antonopoulos^1^

Ms Sheena Thomas^1^

Ms Maria Lyasheva^1^

Dr Evangelos Oikonomou^1^

Dr Nikant Sabharwal^2^

Dr Cheerag Shirodaria^2^

Dr Susan Anthony^2^

Dr Andrew Kelion^2^

Prof Adrian Banning^2^

Dr Rafail Angelos Kotronias^1,2^

Dr Cheng Xie^1,2^

Dr Rajesh Kumar Kharbanda^1,2^

Dr Attila Kardos^1,4^

Dr David Adlam^3^

Dr Amrita Bajaj^3^

Dr Intrajeet Das^3^

Dr Aparna Deshpande^3^

Dr Praveen Rao^3^

Dr Tarun Mittal^5^

Dr Saeed Mirsadraee^5^

Dr Edward Nicol^5^

Dr Jonathan Rodrigues^6^

Dr Benjamin Hudson^6^

Prof John Greenwood^7^

Prof Colin Berry^8,9^

Prof Stephan Achenbach^10^

Dr Mohamed Marwan^10^

Dr Milind Y Desai^11^

Dr Nicholas Screaton^12^

Dr Pál Maurovich-Horvat^13^

Prof Guo-Wei He^14^

Dr Wen-Hua Lin^14^

Dr Li-Juan Fan^14^

Prof Naohiko Takahashi^15^

Dr Hidekazu Kondo^15^

Dr Neng Dai^16^

Prof. Junbo Ge^16^

Prof Bon-Kwon Koo^17^

Dr Gianluca Pontone^18^

Marco Guglielmo^18^

Prof Ron Blankstein^19,20^

Prof Theodora Benedek^21^

Dr Ronak Rajani^22^

Dr Mak Sze Mun^22^

Dr Giulia Benedetti^22^

Dr Rebecca Louise Preston^22^

Dr Elisa McAlindon^23^

Dr Shahzad Munir^23^

Dr Derek Leslie Connolly^24^

Dr William Bradlow^25,26^

Dr Matthias Schmitt^27,28^

Dr Fabiano Serfaty^29^

Dr Ilan Gottlieb^30^

Prof Mario Fritsch T. Neves^31^

Prof David Ernest Newby^32^

Prof Steffen E. Petersen^33,34^

Dr Francesca Pugliese^35^

Dr Nehal N. Mehta^36^

Prof Stéphane Hatem^37^

Prof Alban Redheuil^38^

Dr Georgios Benetos^39^

Prof Meinrad Beer^40^

Dr Gastón A. Rodríguez-Granillo^41^

Prof Joseph Selvanayagam^42^

Prof Bernard Gersh^43^

Dr Francisco Lopez-Jimenez^43^

Dr Ruben De Bosscher^44^

Dr Alain Tavildari^45^

Prof Gemma Figtree^46^

Dr Ibrahim Danad^47^

Dr Ronney Shantouf^48^

Dr Bas Kietselaer^49^

Prof Dimitris Tousoulis^50^

Prof George Dangas^51^

Prof Stefan Neubauer^1^

Prof John Deanfield^52,53^

Prof Keith Channon^1^

Investigator Affiliations:

1. Division of Cardiovascular Medicine, Radcliffe Department of Medicine, University of Oxford, UK
2. Oxford University Hospitals NHS Foundation Trust, Oxford, UK
3. Department of Cardiovascular Sciences and National Institute of Health Research Leicester Biomedical Research Centre, University of Leicester, UK
4. Milton Keynes University Hospital NHS Foundation Trust, UK
5. Royal Brompton and Harefield NHS Foundation Trust, UK
6. Royal United Hospitals Bath NHS Foundation Trust and Department of Health, University of Bath, UK
7. Leeds Teaching Hospitals NHS Foundation Trust, UK
8. NHS Greater Glasgow and Clyde, NHS Scotland, UK
9. Golden Jubilee National Hospital, NHS Scotland, UK
10. Department of Cardiology, Friedrich-Alexander-Universität Erlangen-Nürnberg, Erlangen, Germany
11. Cleveland Clinic Heart and Vascular Institute, Cleveland, USA
12. Royal Papworth Hospital NHS Trust, Cambridge, UK
13. Department of Radiology, MTA-SE Cardiovascular Imaging Research Group, Budapest, Hungary
14. TEDA International Cardiovascular Hospital, Tianjin, China
15. Oita University, Japan
16. Fudan University, China
17. Seoul National University, Seoul, South Korea
18. Centro Cardiologico Monzino IRCCS, University of Milan, Italy
19. Harvard Medical School, Boston, USA
20. Brigham and Women’s Hospital, Boston, USA
21. University of Medicine and Pharmacy of Tirgu Mures, Romania
22. Guy’s and St Thomas’ NHS Foundation Trust, UK
23. Heart and Lung Centre, New Cross Hospital, Wolverhampton, UK
24. Sandwell and West Birmingham Hospitals NHS Trust, UK
25. University Hospitals Birmingham (UHB) NHS Trust, UK
26. University of Birmingham, UK
27. University Hospital of Manchester Foundation Trust, Manchester, UK
28. University of Manchester, Manchester, UK
29. Serfaty Clinicas, Rio de Janeiro, Brazil
30. Casa de Saúde São José, Rio de Janeiro, Brazil
31. Universidade do Estado do Rio de Janeiro, Rio de Janeiro, Brazil
32. University of Edinburgh, Royal Infirmary, Edinburgh, UK
33. The William Harvey Research Institute, Barts and The London School of Medicine and Dentistry, Queen Mary University of London, UK
34. Bart’s Heart Centre, Barts Health NHS Trust, UK
35. NIHR Barts Cardiovascular Biomedical Research Centre, the William Harvey Research Institute, Queen Mary University of London and Barts Health NHS Trust, UK
36. National Institutes of Health, National Heart, Lung, and Blood Institute, USA
37. Foundation for Innovation in Cardiometabolism and Nutrition (ICAN), Paris, France
38. Sorbonne Université, Faculté de Médecine Pierre et Marie Curie, La Pitié Salpêtrière AP-HP, Paris, France
39. Lefkos Stavros Clinic Athens, Greece
40. Universitätsklinikum Ulm, Germany
41. ENERI Medical Institute, Buenos Aires, Argentina
42. South Australia Health and Medical Research Institute, Adelaide, Australia
43. Mayo Clinic, Rochester, USA
44. University Hospitals Leuven, Belgium
45. Cardiovista, France
46. University of Sydney, Australia
47. Amsterdam University Medical Centers, Netherlands
48. Cleveland Clinic Abu Dhabi, UAE
49. Zuyderland Medical Centre, Heerlen, Netherlands
50. University of Athens, Greece
51. Mount Sinai School of Medicine, New York, USA
52. University College London, UK
53. National Institute of Cardiovascular Outcomes Research (NICOR), UK

**Supplemental Figure 1. Preliminary internal validation of the deep-learning model for EAT volume segmentation**

Following initial training of the model but prior to 3 iterations of feedback learning the model was assessed for preliminary performance against human expert segmentation on unseen CCTA. The scatterplot (A) and Bland-Altman plot (B) demonstrate the variability in EAT volume quantification between automated deep-learning machine and human expert analyst.


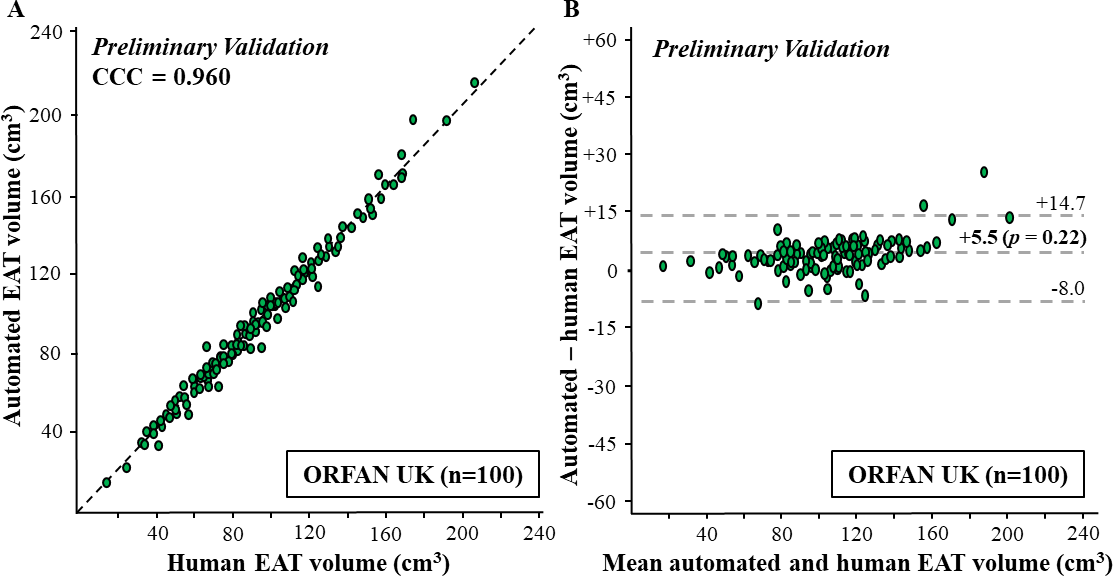


**Supplemental Figure 2. Preliminary internal validation of machine learning model for whole heart segment volume assessment**

Following the initial training of the model and prior to 3 iterations of feedback learning the model was assessed for performance against human expert segmentation in unseen CCTAs. The scatterplot (A) and Bland-Altman plot (B) demonstrate minimal variability in whole heart volume quantification between automated deep-learning machine and human expert analyst. Yellow cases indicate that the analysts indicated they assessed the individual scan to be poor quality. CCC= concordance correlation coefficient; EAT = epicardial adipose tissue; ORFAN = The Oxford Risk Factors And Non Invasive Imaging Study


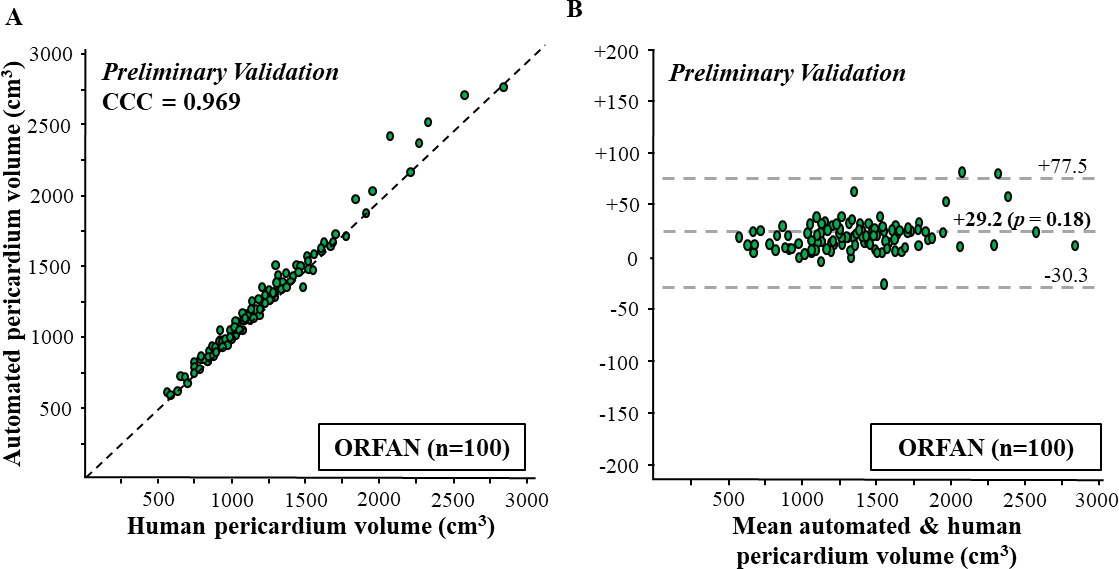


**Supplemental Figure 3. Image of CaRi-Heart user work-view of manual whole heart segmentation**

The whole heart within the bounds of the visceral pericardium is manually segmented incorporating all voxels inferior to the bifurcation of the pulmonary trunk and superior to the most inferior portion of the apex of the heart. Image shows axial (red), coronal (yellow), and sagittal (green) views. Blue = segmented region.


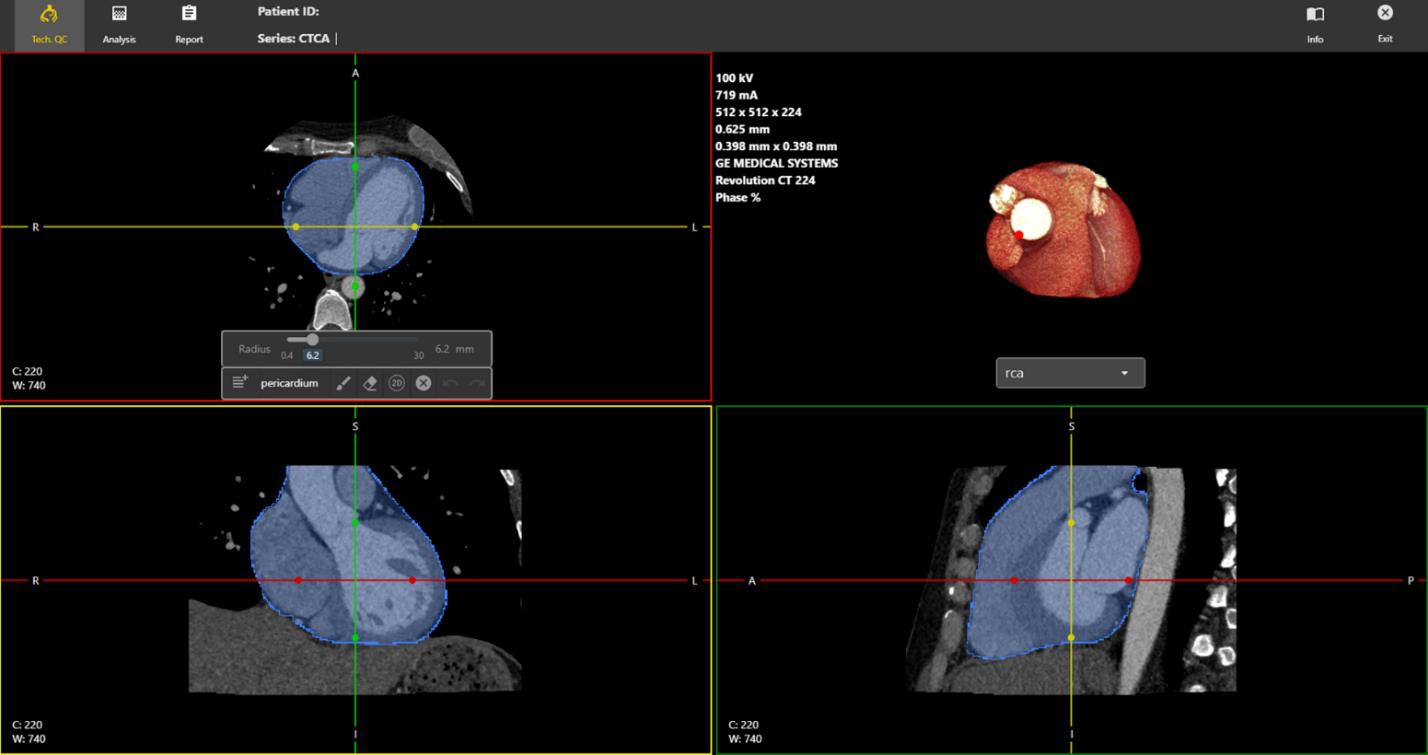


**Supplemental Figure 4. Cross-sectional and longitudinal associations between EAT volume and clinical outcomes in the SCOT-HEART trial with statistically selected risk factor adjustment**

Plots of cross-sectional adjusted risk models for diagnosed AF at the time of the CCTA, adjusted for age, sex, BMI, presence of hypertension, previous heart surgery, presence of valvular heart disease and presence of diabetes (A), and obstructive CAD from CCTA (any 1 coronary vessel with ≥50% stenosis on CCTA), adjusted for age, sex, BMI, presence of hypertension, CAC score, non–high-density lipoprotein cholesterol, and active smoking (B). Odds ratio is shown per SD increase in EAT volume for 1,558 patients randomized to receive CCTA in the SCOT-HEART trial. Plots of longitudinal HRs per SD increase in EAT volume in 1,558 patients randomized to receive CCTA in the SCOT-HEART trial are shown for all-cause mortality (C), and noncardiac mortality (D), both with adjustment for age, sex, BMI, presence of hypertension and diabetes, with further adjustment for active smoking in 6C. Myocardial infarction (E) is shown with adjustment for of age, sex, BMI, presence of hypertension, presence of diabetes, non–high-density lipoprotein cholesterol, obstructive CAD from CCTA (any 1 coronary vessel with ≥50% stenosis on CCTA), and coronary artery calcium score, and stroke (F) is shown with adjustment for age, sex, BMI, presence of hypertension, presence of valvular heart disease and previous heart surgery. AF: atrial fibrillation; BMI: body mass index; CAC: coronary artery calcium score; EAT: epicardial adipose tissue; MI: myocardial infarction; Non-HDL C.: non–high-density lipoprotein cholesterol.


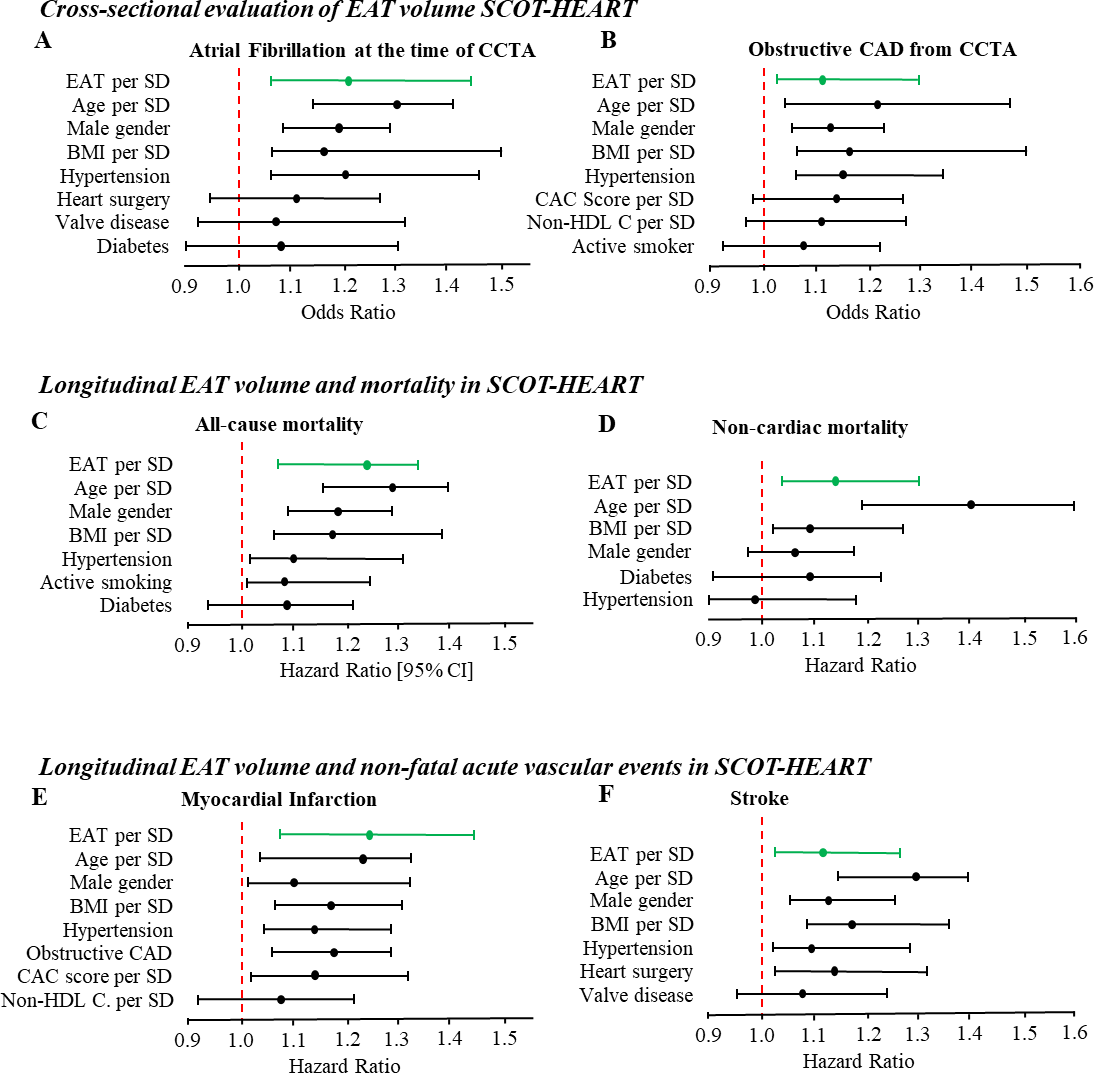


**Supplemental Figure 5. EAT segmentation and whole heart volume assessment between human experts**

The scatterplot (A,C) and Bland-Altman plot (B,D) demonstrate variability in EAT segmentation (A,B) and whole heart volume segmentation (C,D) between each human analyst. CCC= concordance correlation coefficient; EAT = epicardial adipose tissue; ORFAN = The Oxford Risk Factors And Non Invasive Imaging Study.


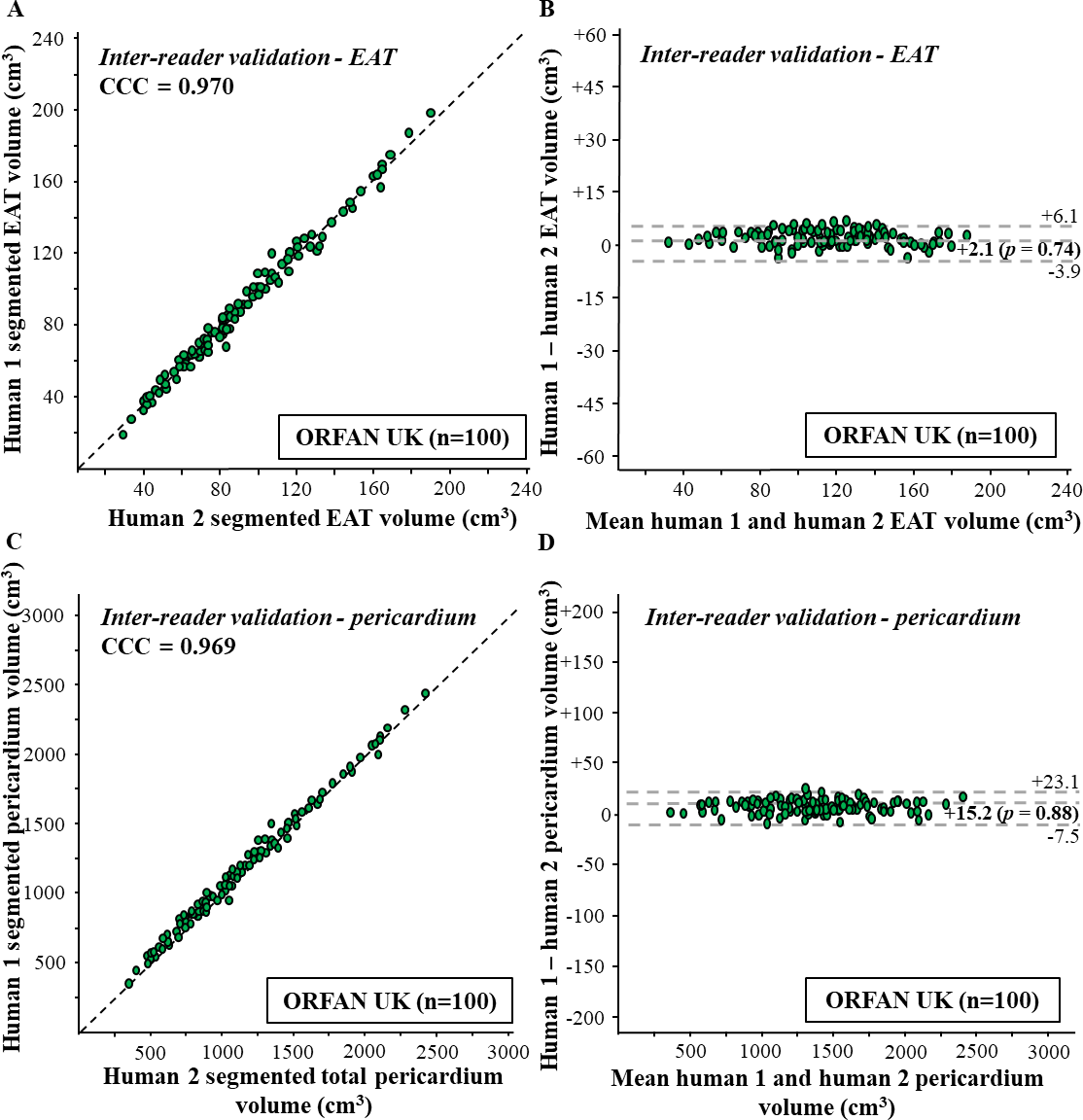


**Supplemental Figure 6. Prognostic value of EAT volume for post-operative AF with adjustment for AF specific risk factors**

Kaplan-Meier curve and adjusted HR for in-hospital postoperative AF (A) and long-term postoperative AF (B), with sample dichotomized by Youden’s J index derived cutpoint of EAT volume (high risk ≥198.7 cm^3^; low risk <198.7 cm^3^), expressed per SD increase of EAT volume. Adjustment is made for age, sex, previous AF diagnosis, presence of hypertension, presence of heart failure, NT-proBNP, previous myocardial infarction, BMI, and LA volume. (C,D) Time-dependent ROC curves for discrimination of in-hospital postoperative AF (C) and long-term postoperative AF (D). CCTA derived LA volume (yellow) is shown alone; model 1 (red) consists of age, sex, previous AF, presence of hypertension, presence of heart failure, NT- proBNP, LA volume, previous MI, and BMI. The addition of EAT volume into model 1 is demonstrated (blue).

AF: atrial fibrillation; AUC: area under curve; BMI: body mass index; EAT: epicardial adipose tissue.


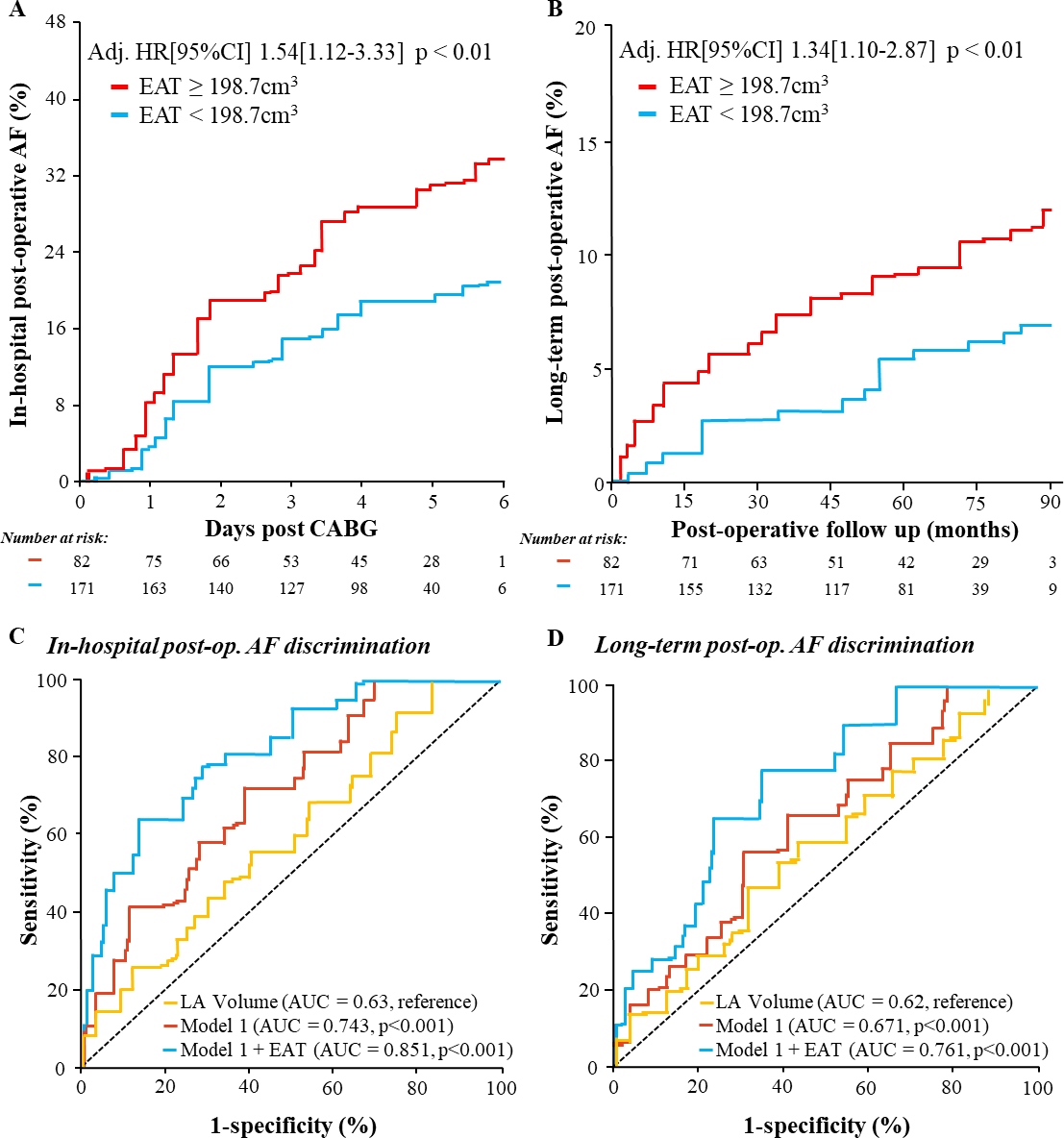


**Supplemental Figure 7. Prognostic value of EAT volume for postoperative AF with adjustment for CVD risk factors including waist-hip ratio**

Kaplan-Meier curve and adjusted HR for in-hospital postoperative AF (A) and long-term postoperative AF (B), with sample dichotomized by Youden’s J index derived cutpoint of EAT volume (high risk ≥198.7 cm^3^; low risk <198.7 cm^3^), expressed per SD increase of EAT volume. Adjustment is made for age, sex, hypertension, diabetes, CAC score and waist-hip ratio. (C,D) Time-dependent ROC curves for discrimination of in-hospital post operative AF (C) and long-term postoperative AF (D). CCTA derived LA volume (yellow) is shown alone; model 1 (red) consists of age, sex, hypertension, diabetes, CAC score and waist- hip ratio. The addition of EAT volume into model 1 is demonstrated (blue).

AF: atrial fibrillation; AUC: area under curve; EAT: epicardial adipose tissue.


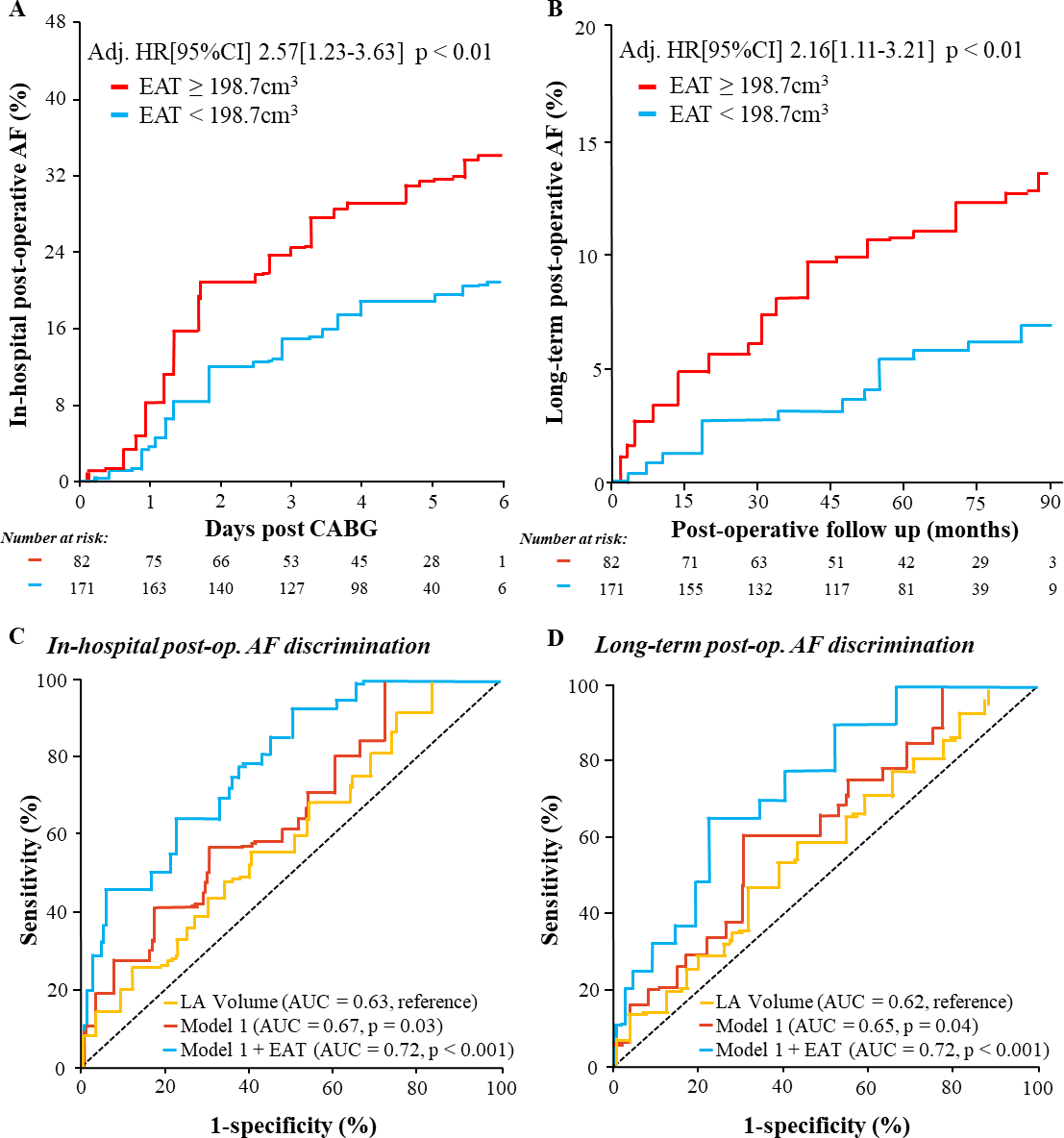


**Supplemental References**

1. SCOT-HEART Investigators. CT coronary angiography in patients with suspected angina due to coronary heart disease (SCOT-HEART): An open-label, parallel-group, multicentre trial. *Lancet*. 2015;385:2383-2391.

2. SCOT-HEART Investigators. Coronary CT angiography and 5-year risk of myocardial infarction. *N Engl J Med.*. 2018;379:924-933.

3. James PA, Oparil S, Carter BL, Cushman WC, Dennison-Himmelfarb C, Handler J, et al. 2014 Evidence-based guideline for the management of high blood pressure in adults: Report from the panel members appointed to the Eighth Joint National Committee (JNC 8). *JAMA*. 2014;311:507-520.

4. Diagnosis and classification of diabetes mellitus. *Diabetes Care*. 2014;37 Suppl 1:S81-90.

5. Stone NJ, Robinson JG, Lichtenstein AH, Bairey Merz CN, Blum CB, Eckel RH, et al. 2013 ACC/AHA guideline on the treatment of blood cholesterol to reduce atherosclerotic cardiovascular risk in adults: A report of the American College of Cardiology/American Heart Association Task Force on Practice Guidelines. *J Am Coll Cardiol*. 2014;63:2889-2934.

6. Newby DE, Williams MC, Flapan AD, Forbes JF, Hargreaves AD, Leslie SJ, et al. Role of multidetector computed tomography in the diagnosis and management of patients attending the rapid access chest pain clinic, the Scottish computed tomography of the heart (SCOT-HEART) trial: Study protocol for randomized controlled trial. *Trials*. 2012;13:184.

7. Newby DE, Adamson PD, Berry C, Boon NA, Dweck MR, Flather M, et al. Coronary CT angiography and 5-year risk of myocardial infarction. *N Engl J Med*. 2018;379:924-933.

8. Thygesen K, Alpert JS, White HD, et al. Universal definition of myocardial infarction. *Circulation*. 2007;116:2634-2653.

9. Hicks KA, Tcheng JE, Bozkurt B, Chaitman BR, Cutlip DE, Farb A, et al. 2014 ACC/AHA key data elements and definitions for cardiovascular endpoint events in clinical trials: A report of the American College of Cardiology/American Heart Association Task Force on Clinical Data Standards (writing committee to develop cardiovascular endpoints data standards). *J Am Coll Cardiol*. 2015;66:403-469.

10. Cutlip DE, Windecker S, Mehran R, Boam A, Cohen DJ, van Es GA, et al. Clinical end points in coronary stent trials: A case for standardized definitions. *Circulation*. 2007;115:2344-2351.

11. Kim WH, Kim CG, Kim D-W. Optimal CT number range for adipose tissue when determining lean body mass in whole-body F-18 FDG PET/CT studies. *Nucl Med Mol Imaging*. 2012;46:294-299.

12. Kvist H, Sjöström L, Tylén U. Adipose tissue volume determinations in women by computed tomography: Technical considerations. *Int J Obesity*. 1986;10:53-67.

13. Mancio J, Azevedo D, Saraiva F, Azevedo AI, Pires-Morais G, Leite-Moreira A, et al. Epicardial adipose tissue volume assessed by computed tomography and coronary artery disease: A systematic review and meta-analysis. *Eur Heart J Cardiovasc Imaging*. 2017;19:490-497.

14. Oikonomou EK, Marwan M, Desai MY, Mancio J, Alashi A, Hutt Centeno E, et al. Non-invasive detection of coronary inflammation using computed tomography and prediction of residual cardiovascular risk (the CRISP CT study): A post hoc analysis of prospective outcome data. *Lancet*. 2018;392:929-939.

15. Antonopoulos AS, Sanna F, Sabharwal N, Thomas S, Oikonomou EK, Herdman L, et al. Detecting human coronary inflammation by imaging perivascular fat. *Sci Transl Med.* 2017;9:eaaal.
